# CO_2_ electroreduction on Cu operates via an alternative chain growth mechanism to form C–C bonds at elevated temperature and pressure

**DOI:** 10.1038/s41929-025-01451-1

**Published:** 2025-12-12

**Authors:** Rafaël E. Vos, Pengfei Sun, Daniel Schauermann, Hassan Javed, Selwyn R. Hanselman, Gang Fu, Marc T. M. Koper

**Affiliations:** 1 Leiden Institute of Chemistry, Leiden University, Leiden, Netherlands; 2 State Key Laboratory of Physical Chemistry of Solid Surfaces, Collaborative Innovation Center of Chemistry for Energy Materials, National Engineering Laboratory for Green Chemical Productions of Alcohols, Ethers and Esters, College of Chemistry and Chemical Engineering, Xiamen University, Xiamen, China

**Keywords:** Electrocatalysis, Electrocatalysis

## Abstract

Future practical applications of the electrochemical CO_2_ reduction reaction will probably involve the use of higher pressures and temperatures. However, most research on the copper-catalysed electrochemical CO_2_ reduction reaction—the most widely studied system due to its C–C coupling ability—is typically performed under ambient conditions, and hence the mechanistic conclusions drawn also pertain to those conditions. Using a custom high-pressure, high-temperature electrochemical cell, we show here that on copper electrodes, the C–C coupling mechanism changes from the typical CO dimerization mechanism at low temperatures to a Fischer–Tropsch-like chain growth mechanism at temperatures above 125 °C (also requiring higher pressure). These results show that temperature and pressure are crucial parameters to consider in applied and mechanistic studies of the electrochemical reduction of CO_2_ because they can open up alternative reaction pathways and alter known mechanisms.

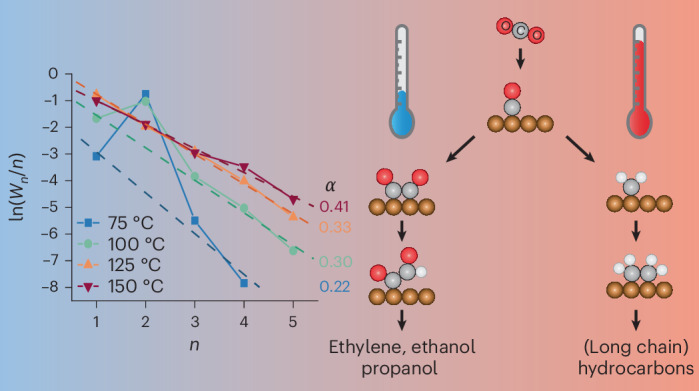

## Abstract (web summary)

Electrocatalytic CO_2_ reduction is typically studied at laboratory scale under ambient conditions; however, temperature and pressure may have a profound impact on the mechanism of this reaction and on its relevance to industrial applications. This study uses a custom temperature- and pressure-adjustable cell to reveal a chain growth mechanism emerging on copper electrodes at elevated temperatures and pressures.

## Main

The electrochemical CO_2_ reduction reaction (CO_2_RR) offers an interesting strategy to recycle CO_2_ into fuels and chemicals using renewable electricity^1–4^. Copper is a unique catalyst for this reaction because it is the only catalyst that can make C–C bonds with significant Faradaic efficiencies (FEs)^5–7^. Multicarbon products in the CO_2_RR are typically formed via a CO dimerization or carbonyl coupling mechanism^8–13^, although recent research has provided evidence for a chain growth mechanism as an alternative C–C coupling mechanism, particularly for nickel electrodes^14–17^. Many factors influencing the CO_2_RR on copper have been studied, such as morphology^18–21^, electrolyte effects^22–25^ and electrode potential^7,11,24–26^. Recently, more attention has been given to parameters such as pressure^27–30^ and temperature^14,31–34^. Electrochemistry research in general, and CO_2_RR studies specifically, are often performed at ambient pressure and temperature, and hence conclusions and fundamental insights are drawn for these conditions. However, for practical applications, ambient conditions might not be the most relevant because industrial electrolysers typically operate at elevated temperatures^35^, for example, due to thermal losses^36–38^ and/or hot feedstocks. Moreover, pressure and temperature can improve the selectivity and activity of the CO_2_RR^27–29,31,32^, and open up different mechanistic pathways^30,39^, and elevated pressure could enable better integration with up- and downstream processes^30,40,41^.

Temperature has a significant effect on the activity and selectivity of the CO_2_RR on copper at ambient pressure^32^. Below ∼48 °C, increasing temperature has a positive effect on both the selectivity and activity towards C_2+_ products, but above 48 °C the competing hydrogen evolution reaction (HER) takes over. Other studies^27–30,40,42–44^ have shown that increased CO_2_ pressures can suppress the HER, which might mean that the optimum in C_2+_ selectivity with temperature changes at elevated pressures. Moreover, at elevated pressures, the studied temperature window can be increased and experiments in aqueous electrolytes can be performed above 100 °C. This could bridge the fields of electrocatalysis and thermal catalysis, where C–C bonds are made via a chain growth mechanism in the Fischer–Tropsch reaction^45–47^ (although copper produces mainly methanol in thermal heterogeneous catalysis^48–50^). This raises the question whether electrochemical CO_2_ reduction on copper at high temperatures in aqueous electrolyte is more similar to electrocatalysis at ambient conditions, to thermal catalysis, or to neither of them. Recently, we have developed an electrochemical cell in which both temperature and pressure can be regulated up to 200 °C and 140 bar, respectively^51^.

In this study, we use this set-up to investigate the combined effect of elevated pressure and temperature to show a change in the C–C coupling mechanism on copper electrodes above 100 °C, at a pressure of 24 bar. Whereas at low temperatures C–C bonds are mostly formed via the traditional CO dimerization or carbonyl coupling mechanism, at high temperatures the chain growth mechanism takes over. From 125 °C and higher, this is the dominant mechanism making C–C bonds. As supported by density functional theory (DFT) calculations, we attribute this effect to the enhanced C–O bond breaking at higher temperature, leading to CH_*x*_ fragments on the surface which serve as intermediates for Fischer–Tropsch-like chain growth. This study illustrates that temperature and pressure are crucial parameters to consider in applied and fundamental studies of the electrochemical reduction of CO_2_.

## Results

### Experimental results

**Fig. 1:**
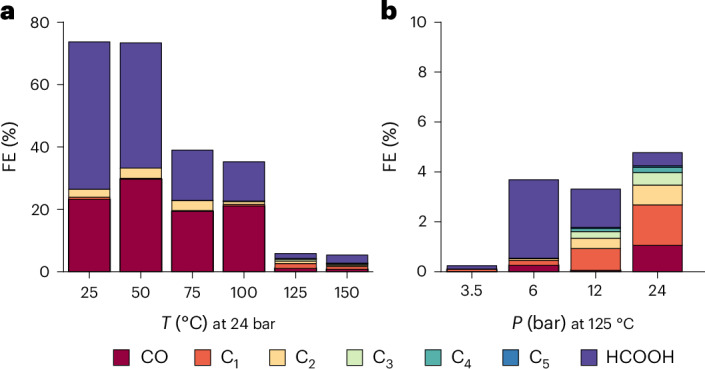
FEs for CO_2_RR products on a copper wire. **a**,**b**, FE as a function of temperature at 24 bar of CO_2_ pressure (**a**) and pressure at 125 °C (**b**), both at −1.3 V versus SHE in 0.2 M KHCO_3_. The C_1_–C_5_ products are only hydrocarbons. The remaining FE goes towards hydrogen as is shown in Supplementary Fig. 2 and Supplementary Table 3.

Figure 1 shows the temperature (25–150 °C) and CO_2_ pressure (3.5–40 bar) dependence of the FE of the CO_2_RR on copper at −1.3 V versus SHE (standard hydrogen electrode). This figure shows the FE for CO, HCOOH and hydrocarbons up to C_5_ (methane, ethane, ethylene, propane, propene, butane, butene, isobutane, isobutane, isopentane, pentane, 1-pentene). The detailed FE per product is shown in Supplementary Tables 3 and 4. Besides small amounts of acetate, no other CO_2_RR products have been observed and the remaining FE goes towards H_2_ and is shown in Supplementary Fig. 2 and Supplementary Table 3. A main observation from Fig. 1a is that above 100 °C, mainly hydrogen is produced, with only small amounts of CO_2_RR products remaining. Figure 1b shows that pressure mitigates this effect only to some extent. The potential of −1.3 V versus SHE was chosen because copper is very unstable at higher overpotentials at temperatures above 100 °C, as can be seen in Supplementary Fig. 1. At −1.5 V versus SHE copper only produces H_2_ within the total experiment time (Supplementary Fig. 2b). However, at lower overpotentials some CO_2_RR is still observed, even at 150 °C. Besides the dominant H_2_ production (Supplementary Fig. 2), the product distribution changes from mainly CO and HCOOH to hydrocarbons. As mentioned, pressure also influences the CO_2_RR selectivity compared to the HER at 125 °C, with higher pressures leading to higher CO_2_RR rates and a more stable operation, as illustrated in Supplementary Fig. 1a. The selectivity of the CO_2_RR products changes as well because higher pressures lead to more CO and hydrocarbons, and to less HCOOH. The selectivity has a less clear trend with potential, although a potential of −1.3 V versus SHE seems to be the optimum for hydrocarbon formation, as shown in Supplementary Fig. 2b.

**Fig. 2:**
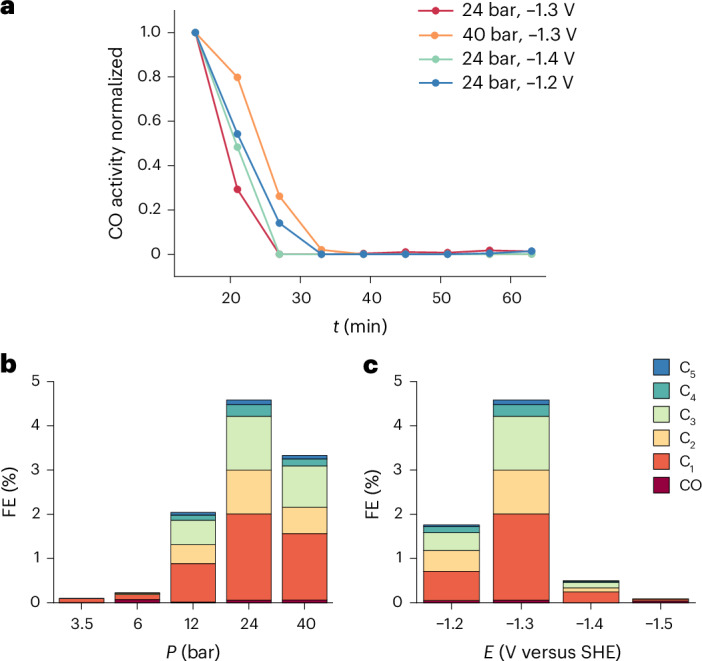
Time effect on the selectivity of the CO_2_RR at 125 °C. **a**, Normalized CO activity as function of time for several conditions, all at 125 °C. This illustrates that CO is only produced at the start of the experiment because it decreases rapidly and from 32 min the CO production is negligible. **b**,**c**, Partial current density for the CO_2_RR products but only taking into account the gaseous products during the last 30 min of the experiment, as a function of pressure at 125 °C and −1.3 V versus SHE (**b**) and potential at 24 bar and 125 °C (**c**).

Interestingly, the CO produced at high temperatures is mainly produced at the start of the experiment, as shown in Fig. 2a, while the hydrocarbons are produced throughout the measurement. This observation coincides with a significant change in the current at the start of the experiment, after which it stabilizes, as shown in Supplementary Fig. 1b. It is possible that certain active sites are blocked or opened up, changing the CO_2_RR product during this initial period. Another possibility is that the restructuring of the copper surface at these elevated temperatures, as we have observed on copper in a previous study^32^, leads to this change in current and selectivity during this initial period. The time dependence of the restructuring requires further study, which is challenging, however, in this high-temperature, high-pressure set-up because it requires extra time to release the pressure and reduce the temperature before the copper electrode can be studied after stopping the experiment, and no in situ characterization or spectroscopy is currently possible. We performed X-ray photoelectron spectroscopy on the copper electrode after the experiment to test if a significant amount of metal or any other deposit exists on the surface. No metal signal other than copper was observed (Supplementary Fig. 6), so this can also not explain the transient CO production. Furthermore, this experiment proves that iron (from the steel vessel) is not responsible for the observed reactivity.

When only the gaseous products of the last 30 min are analysed, almost no CO is produced and hydrocarbon selectivity slightly increases. The optimum for the hydrocarbon formation also becomes more apparent, as illustrated in Fig. 2. High overpotentials are needed to drive the chain growth mechanism, but overpotentials that are too high apparently destabilize the copper surface, resulting in lower chain growth activity. Moreover, high pressures are needed to stabilize the copper at these high temperatures and therefore the FE towards the hydrocarbons increases with pressure. However, the FE at the highest pressure of 40 bar seems to decrease again slightly compared to the FE at 24 bar, which might be due to blocking of active sites for the chain growth mechanism by adsorbed CO_2_ or CO^51^.

**Fig. 3:**
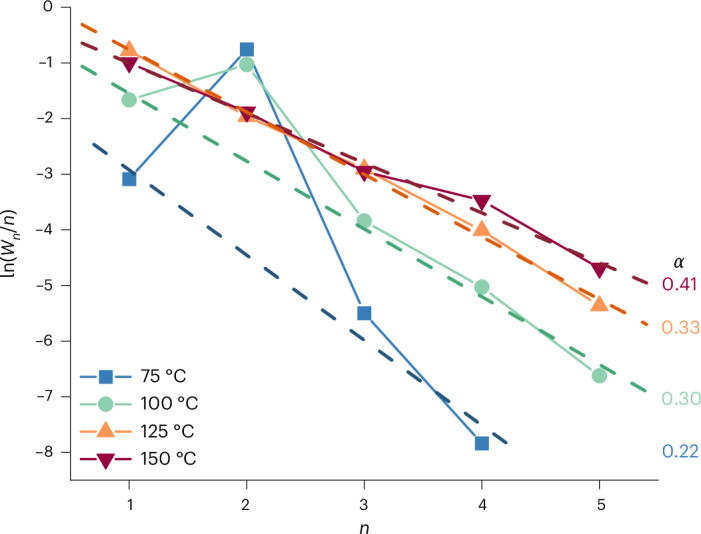
Increase in chain growth probability on copper with temperature. ASF plot (dotted lines) for copper at −1.3 V versus SHE at 24 bar in 0.2 M KHCO_3_ as a function of temperature. The slope of the graph gives the chain growth probability *α* as shown on the right and in Supplementary Table 4. *W*_*n*_ is the weight fraction, and *n* is the chain length.

Copper is known to be able to form C–C bonds and produce C_2+_ products. However, longer-chain hydrocarbons are usually not observed on copper. Either C_1_ (methane) or C_2_ (ethylene) hydrocarbons are formed, or C_3_ oxygenates, such as propionaldehyde and 1-propanol. In rare instances higher hydrocarbons have been observed before on Cu^52–54^ although they are more often found on other CO_2_RR catalysts^16,17,55–58^, with the highest selectivities on nickel^14,15^. These longer hydrocarbons are presumed to be formed via a CH_*x*_ chain growth mechanism^15,57^ similar to the thermal catalytic Fischer–Tropsch reaction, while copper traditionally forms C–C bonds via a CO dimerization (pathway C–D–E in Fig. 7) or a carbonyl coupling mechanism (pathway F–G–E). Figure 1a shows that at −1.3 V versus SHE (standard hydrogen electrode) at low temperatures, mainly CO and HCOOH are formed, but also some ethylene is formed via the CO dimerization mechanism. When the temperature increases, longer-chain products are detected and from 75 °C onwards an Anderson–Schulz–Flory (ASF) plot can be constructed because C_4_ products such as butene and butane are observed. From the ASF plot in Fig. 3, it can be seen that at 75 and 100 °C, two mechanisms are active in forming C–C bonds on copper. One is the chain growth mechanism previously observed on nickel, resulting in a linear relationship in the ASF plot. However, the C_2_ products do not fit this linear relationship because they are also produced via the CO dimerization mechanism, which produces mainly ethylene, leading to a peak at *n* = 2 in the ASF plot. At 125 and 150 °C, the product distribution fits the linear relationship in the ASF plot, including the C_2_ products, which shows that at these temperatures, all hydrocarbons are formed via the chain growth mechanism and the CO dimerization/carbonyl coupling mechanism has become inactive.

From the slope of the linear relationship in the ASF plot, the chain growth probability can be determined. The chain growth probability gives an indication for the product distribution, where a chain growth probability of 0 leads to only methane and a chain growth probability close to 1 leads to very long hydrocarbons consisting of >20 carbon atoms. Figure 3 shows that the chain growth probability on copper increases with increasing temperature. Supplementary Table 5 shows that pressure and potential also seem to influence the chain growth probability, decreasing with increasing pressure and higher overpotential.

**Fig. 4:**
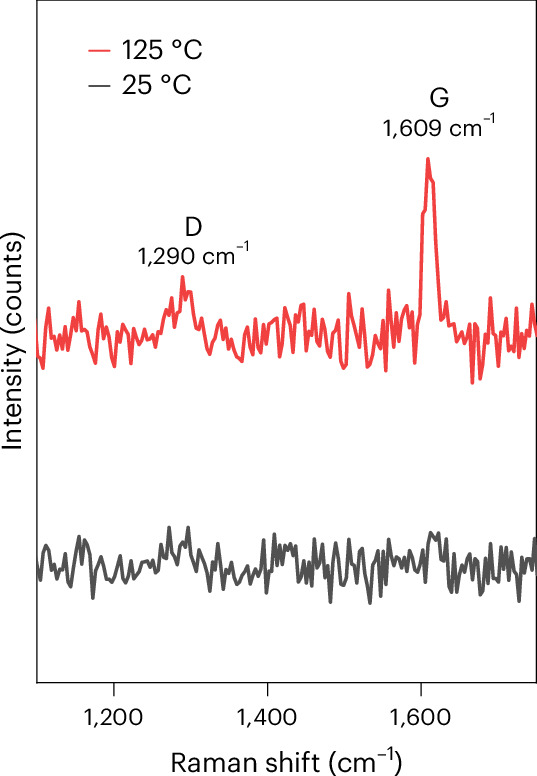
Raman spectroscopy on copper catalysts after CO_2_RR. Raman spectra after 1 h of the CO_2_RR on copper in 0.2 M KHCO at 25 and 125 °C, 24 bar. The graph shows the formation of graphite-like carbon species (coke) on the electrode at elevated temperatures.

Although the above results show a clear change in mechanism towards chain growth, they also demonstrate that H_2_ is the primary product on copper at high temperature. On nickel, which is a chain growth catalyst at room temperature, it has been shown by Raman spectroscopy that the electrocatalyst is deactivated by coke deposits. These graphite-like carbon species form on the catalyst surface during the chain growth mechanism^14^ and are considered poisons for the formation of hydrocarbons^59–61^. Coke-covered nickel is still active for the HER but inhibits chain growth during the CO_2_RR. Therefore, we performed ex situ Raman spectroscopy on copper catalysts after 1 h of electrolysis at 25 and 125 °C, similar to experiments in Fig. 1a. Figure 4 suggests that coke deposits are also formed on copper at high temperatures. A G peak around 1,609 cm^−1^ can be clearly observed at 125 °C and also a small D peak is observed at this temperature, whereas at 25 °C no such features can be distinguished. The extended spectra in Supplementary Fig. 7 show that on both samples a copper oxide feature is visible, indicating that the lack of coke features in the 25 °C sample is not due to general signal loss. The fact that the G peak is clearer than the D peak for the sample at 125 °C agrees with the experiments on nickel^14^, where the G peak is more intense than the D peak, indicating that the coke formed during the chain growth mechanism is primarily graphite-like carbon. However, on copper the features are not as intense as previously observed on nickel, suggesting that less coke is formed on copper than on nickel. Nevertheless, we hold this graphitic-like carbon formation responsible for the extensive H_2_ formation under conditions where copper forms hydrocarbons. We also note that this deactivation of the CO_2_RR at high temperature and high pressure was not observed on gold electrodes, where in fact high pressures give rise to very stable and high FEs of the CO_2_RR to CO (ref. ^51^). We hypothesize that this is related to the lack of carbon deposits on gold.

### DFT calculations

To gain a deeper understanding of the underlying mechanism, we conducted a detailed investigation into the potential decomposition of the three main intermediates (*CO, *CHO, *COH) of CO_2_ electroreduction into *CH_*x*_ species using DFT calculations. The aim of these calculations is to investigate the barrier for C–O dissociation in different possible pathways, and to compare the barrier on two different facets of copper and nickel surfaces.

First, we computed the barrier for C–O dissociation in the above three intermediates (that is, to *C and *O, to *CH and O, and to *C and *OH, respectively), incorporating an implicit solvation model to account for solvent effects. This calculation does not involve proton-coupled electron transfer (PCET), and the influence of electrode potential is not considered here. We constructed models of the Cu (100) and Cu (211) surfaces, which are the predominant crystal facets of polycrystalline copper during CO_2_RRs^62,63^. For comparison, we also constructed models of Ni (100) and Ni (211). These four models are shown in Supplementary Fig. 8. More technical details of the calculations are given in Supplementary Notes 1–6.

**Fig. 5:**
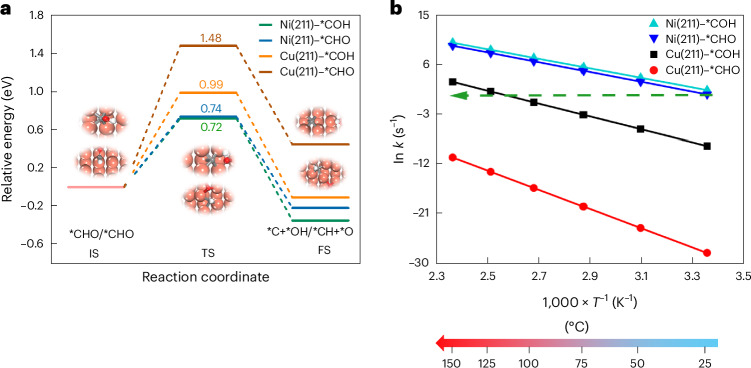
DFT calculations for *COH/*CHO dissociation on copper and nickel. **a**, Profiles for initial states (IS), transition states (TS) and final states (FS) of *COH/*CHO dissociation on Cu (211) and Ni (211) surfaces. Representative structures of the initial, transition and final states of *COH/*CHO dissociation on the Cu (211) surface are also shown. **b**, Variation of dissociation rate constants (*k*) of *COH/*CHO with temperature on Cu (211) and Ni (211) surfaces. The green line represents the dissociation rates of *COH/*CHO on the nickel surface at 25 °C. The calculation of rates is explained in Supplementary Note 3.

First, we considered the possibility of *CO directly dissociating into *C and *O on these four surfaces. Our calculations revealed that the energy barriers for the direct decomposition of *CO on all four surfaces are prohibitively high, being 2.79, 3.00, 1.73 and 2.89 eV on Cu (100), Cu (211), Ni (100) and Ni (211), respectively, as shown in Supplementary Fig. 9. Hence, we consider this an unlikely pathway. Next, we examined the role of hydrogen in assisting the dissociation of *CO, specifically the decomposition of the *COH and *CHO species. On both the (100) and (211) facets, we find that the dissociation of the *COH intermediate has the lowest barrier on both copper and nickel, with barriers generally lower on the (211) facet. Figure 5a shows the energy profile for the dissociation of *COH and *CHO on the Cu (211) surface in comparison to the Ni (211) surface. Specifically, the energy barrier for the decomposition of *COH to *C + *OH is 0.27 eV higher on copper than on nickel, while the barrier for the decomposition of *CHO to *CH + *O is higher by 0.74 eV. Similar results are observed for the Cu (100) and Ni (100) surfaces, as demonstrated in Supplementary Fig. 10a. To quantitatively compare the effect of temperature on the decomposition rate of *COH on the Cu (211) and Ni (211) surfaces, we plotted the reaction rate constants as a function of temperature by using the Arrhenius equation. As shown in Fig. 5b, the dissociation rate of *COH on copper at ∼125–150 °C approaches that of *COH on nickel at room temperature. While this is obviously somewhat coincidental, it illustrates that DFT indeed predicts in a general sense that the chemistry which happens on nickel at room temperature is expected to occur on copper at temperatures in the experimentally observed temperature range. On the (100) surface, the qualitative temperature effect is the same as on (211), although rates are generally predicted to be lower, as shown in Supplementary Fig. 10b.

Second, we performed a calculation for a different mechanism for C–O activation, namely, one in which the oxygen fragment does not adsorb on the copper or nickel surface but splits off as a H_2_O ending up in the electrolyte solution when H^+^ is the proton donor, or as OH^−^ when water is the proton donor. Because protons and electrons transfer during this reaction, it constitutes a PCET process, which is highly sensitive to the applied potential. Therefore, the simulation is performed under constant-potential conditions. Technical details of the simulation are given in the Supplementary Note 4.

**Fig. 6:**
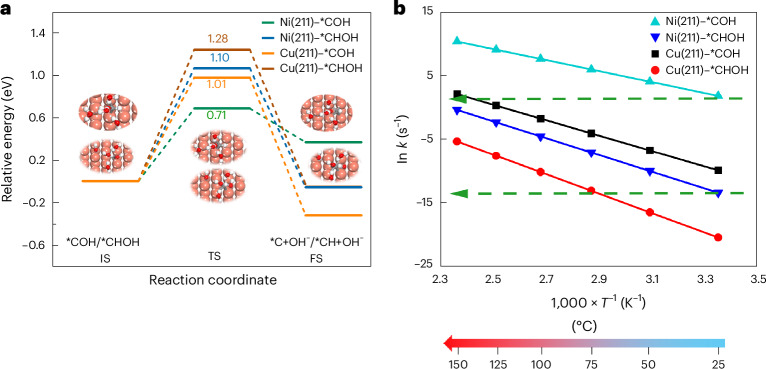
DFT calculations for the *COH/*CHOH PCET process on copper and nickel. **a**, Profiles for initial states (IS), transition states (TS) and final states (FS) of the *COH/*CHOH PCET process on Cu (211), and Ni (211) surface at *U* = −1.3 V versus SHE. Representative structures of the initial, transition and final states of the *COH/*CHOH PCET process on the Cu (211) surface are also shown. **b**, Variation of the PCET process rate constants (*k*) of *COH/*CHOH with temperature on Cu (211) and Ni (211) surfaces. The green line represents the PCET process rates of *COH/*CHOH on the nickel surface at 25 °C. The calculation of rates is explained in Supplementary Note 3.

Specifically, we investigated the formation of *CH_*x*_ species from *COH and *CHOH intermediates via the PCET process at a potential of −1.3 V versus SHE. The energy barrier for the conversion of *COH to *C was higher on Cu (100) (0.99 eV) than on Ni (100) (0.62 eV), and similarly higher on Cu (211) (1.01 eV) than on Ni (211) (0.71 eV). Likewise, the energy barrier for the conversion of *CHOH to *CH was higher on Cu (100) (0.85 eV) than on Ni (100) (0.70 eV), and similarly higher on Cu (211) (1.28 eV) than on Ni (211) (1.10 eV), as shown in Fig. 6a and Supplementary Fig. 11a. The calculations were performed with water as proton source. Using H^+^ as the proton donor results in the same qualitative effect, but with a lower absolute barrier, as shown in Supplementary Figs. 12 and 13. To quantitatively compare the effect of temperature on the *COH/*CHOH PCET process rate on Cu (211) and Ni (211) surfaces, we used the Arrhenius equation to plot the reaction rate constants as a function of temperature. As shown in Fig. 6b, the *COH PCET process rate on Cu (211) at ∼125–150 °C approaches that observed for *COH on Ni (211) at room temperature. The *CHOH PCET process rate on Cu (211) at ∼50–75 °C approaches that observed for *CHOH on Ni (211) at room temperature. On the (100) surface, the qualitative temperature effect is the same as on Cu (211), as shown in Supplementary Fig. 11b.

We further considered the issue of copper oxidation at high temperatures. Our calculations indicate that partially oxidized copper is unfavourable for the formation of CH_*x*_ species due to higher reaction barriers compared to pure copper surfaces, as shown in Supplementary Table 6. Moreover, Pourbaix diagrams (Supplementary Figs. 14 and 15) show that metallic copper should still be the thermodynamically preferred state under the conditions in this study. Hence, any copper oxides during the CO_2_RR should be metastable phases and hence at higher temperatures they are expected to be reduced faster. Therefore, it is not likely that copper oxidation is a factor in the chain growth mechanism.

To further elucidate the pathway for hydrocarbon chain growth, we investigated the coupling of *CH_2_ with various C_1_ intermediates (*CO, *C, *CH, *CH_2_ and *CH₃) on Cu (100) and Cu (211) surfaces via DFT calculations. As summarized in Supplementary Table 7, the coupling of *CH_2_ with another *CH_2_ to form *CH_2_CH_2_ exhibits the lowest activation energy barriers—0.05 eV on Cu(100) and 0.03 eV on Cu(211)—among all considered pathways. These values are nearly barrierless and significantly lower than those for other coupling scenarios, such as *CH_2_ + *CO (0.72 eV and 0.59 eV). Moreover, the formation of *CH_2_CH_2_ is also the most thermodynamically favourable, with reaction energy changes (Δ*E*) of −1.86 eV on Cu(100) and −1.54 eV on Cu(211), further supporting its preference over other routes. These results highlight that the *CH_2_–*CH_2_ coupling is both kinetically and thermodynamically favourable, making it the dominant initial C–C bond formation step in the chain growth process on metallic copper surfaces.

Finally, we investigated the adsorption properties of *COH and *CHO species on copper and nickel to analyse the nature of their difference. Our findings reveal that the adsorption of these two species on copper is weaker compared to that on nickel (Supplementary Fig. 16). A crystal orbital Hamilton population (COHP) analysis was also conducted to evaluate the strength of the internal C–O bonds at the four surfaces. The integrated COHP (ICOHP) values for Cu (211) and Ni (211) were determined to be −8.70 eV, −4.52 eV for the *COH and −9.23 eV and −5.04 eV for the *CHO, respectively. On the (100) surface, copper and nickel exhibit similar trends of ICOHPs (Supplementary Fig. 17). A more negative ICOHP indicates a stronger C–O bond. The ICOHP analysis confirms that the C–O bond in *COH is easier to activate than in *CHO, and that nickel is more suited for activating the C–O bond than copper. Therefore, the adsorption energies and COHP combined indicate that it is more difficult to activate the C–O bond of *COH (and *CHO) on copper than on nickel. This is of course not unexpected because nickel is a less noble metal than copper, with a higher *d*-band position.

The above calculations indicate that, regardless of whether C–O dissociation happens through the hydrogen-assisted *CO dissociation mechanism or the *COH/*CHOH PCET process, the formation of *CH_*x*_ species occurs more readily on nickel than on copper. The difference in activation energy is such that copper is expected to exhibit behaviour similar to nickel at temperatures above 100 °C, thereby enabling *CH_*x*_ coupling to produce long-chain hydrocarbons.

### General mechanism

**Fig. 7:**
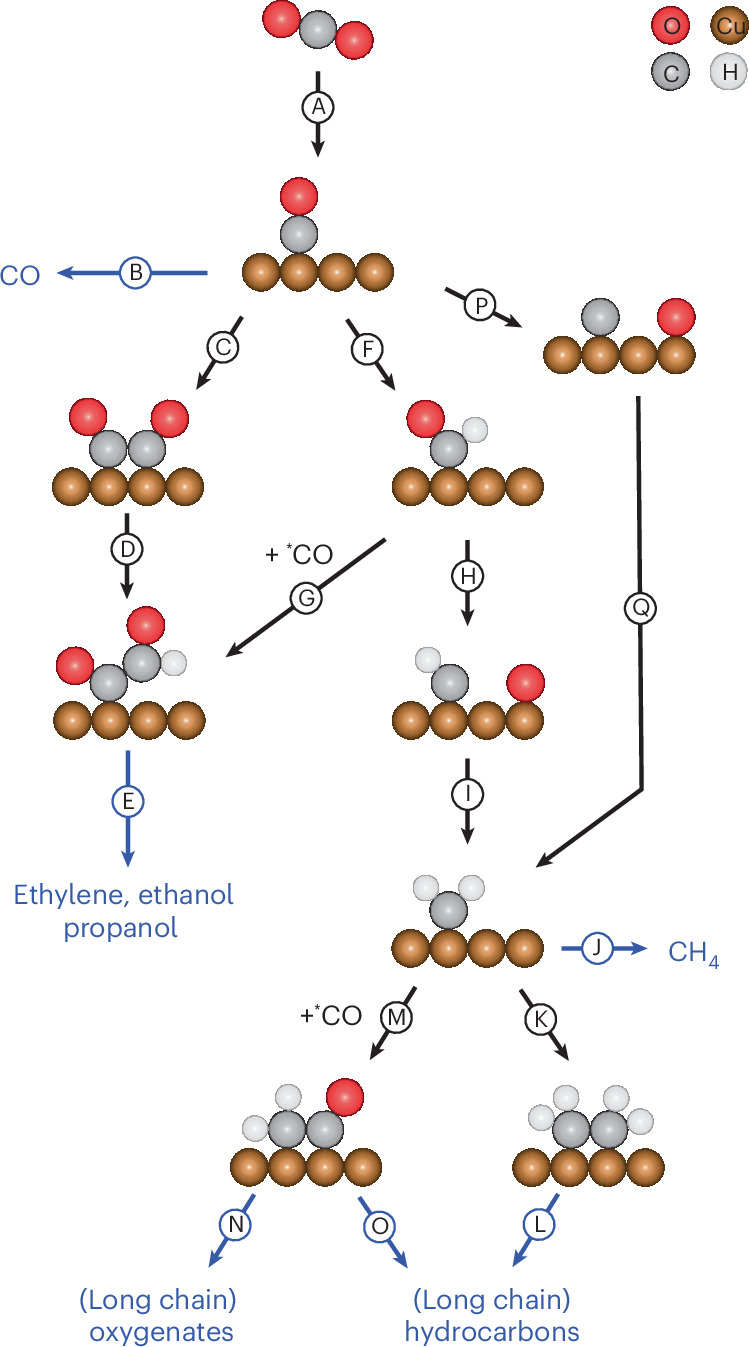
Overview of the different C–C coupling mechanisms on copper. The main final products are shown in blue. Pathways are indicated with an arrow. The number of water molecules and electrons necessary for each pathway are not indicated. Pathway A is the CO_2_ reduction to form *CO, pathway B is the desorption of CO, pathway C is CO dimerization and pathway G is the carbonyl coupling to form the C–C bond. Pathways D, F, I and Q are the further hydrogenation of intermediates. Pathway H is the hydrogen-assisted CO dissociation, pathway K is CH_*x*_ coupling, pathway M is CO insertion, and pathways E, J, L, N and O consist of multiple steps to form the final hydrocarbons and oxygenates.

Figure 7 shows an overview of the different reaction pathways leading to C–C coupled products. At high temperatures the traditional C–C coupling pathways via dimerization (pathway C) or carbonyl coupling (pathway G) do not take place because the intermediates feeding those pathways dissociate into CH_*x*_ and O (or dissolved OH^−^) fragments. Alternatively, the C and G pathways may lose prominence due to a lower *CO coverage (desorption of *CO (pathway B) should be faster at higher temperatures) because the *CO coverage is probably an important determinant for this pathway^64–68^. The chain growth mechanism is expected to take place via a CH_*x*_ intermediate^15,57^ and would be less sensitive to the *CO coverage. However, we observe only little CO as product at high temperatures, and only at the start of the reaction, while hydrocarbon formation still takes place. This indicates that the *CO mostly reacts before it can desorb and favours the explanation involving HCO/COH dissociation.

Hydrogenation is important in the chain growth mechanism, and in all cases where this mechanism is observed during the CO_2_RR, it is under conditions where the HER is dominant and very little CO is formed^14,15,17,56–58^. Hydrogenation of the *CO can take place via a so-called hydrogen-assisted dissociation to form *CH_*x*_ intermediates (pathways F–H–I). Alternatively, in the thermocatalytic Fischer–Tropsch reaction a direct dissociation of *CO is possible^69–71^ (pathway P), which is followed by hydrogenation of the *C to form the *CH_*x*_ intermediates, which we consider unlikely under electrochemical conditions on account of the high activation energy calculated for this step. The hydrogen-assisted dissociation via pathway F, either through chemical dissociation or through a PCET step, is much more likely based on the DFT results. At high temperatures, pathway F should become so fast that pathways B and C are outcompeted.

At low temperatures, methane is formed on copper^7,11,68,72^ via pathways F–H–I–J, but pathways M and K seem not to be accessible because no longer hydrocarbons (apart from ethylene) have been observed. Therefore, for the chain growth mechanism, high temperatures should enhance the C–C coupling compared to CH_4_ formation, as also suggested by the increase in chain growth probability with temperature. The chain can grow either by CH_*x*_ coupling (pathway K) or by CO insertion (pathway M). It is still ambiguous which pathway is followed in the electrochemical chain growth mechanism. However, we argue that on copper at high temperatures the C–C bond is probably formed via CH_*x*_ coupling. If pathway M were active, pathways B and G would also be expected to be active. However, as discussed above, very little *CO is available, making these pathways unlikely. Moreover, pathway M should result in some oxygenates. However, neither long- nor short-chain oxygenates have been detected at high temperatures. In a study on other metal catalysts under similar high-pressure, high-temperature conditions, we found that the chain growth mechanism can occur on other catalysts such as iron and zirconium. Also, in these cases no long- or short-chain alcohols were detected^73^. Furthermore, DFT calculations suggested that *CH_2_–*CH_2_ coupling has a lower barrier than *CH_2_–*CO and other coupling paths. High temperatures should thus open up pathway K and must make this pathway competitive with pathway J (methane formation) to explain our results. Raman or infrared spectroscopy would be excellent tools to gain more insights into the mechanism; however, this is currently not possible with our set-up. The development of a high-temperature, high-pressure electrochemical cell with a window or a probe to perform spectroscopy would be needed for this.

## Conclusions

We have investigated the combined effect of pressure and temperature on the electrochemical CO_2_RR on copper. We have shown that temperature has a larger effect on the selectivity than pressure, although the effect of the latter is not negligible. Elevated pressures allow the operation of aqueous CO_2_RRs at temperatures above 100 °C. We demonstrated CO_2_RR experiments at these high temperatures with liquid electrolytes. Although the HER dominates at these conditions, we observed a prominent change in the C–C coupling mechanism. Traditionally, C–C bonds are believed to form through a CO dimerization mechanism on copper, at least at room temperature. However, by increasing the temperature, a Fischer–Tropsch-like chain growth mechanism, in which higher hydrocarbon chains are formed, becomes more active, and becomes dominant at temperatures higher than 125 °C.

## Methods

### Chemicals

The electrolyte was prepared from KHCO_3_ (99.95%, Sigma-Aldrich) with Milli-Q water (≥18.2 MΩ·cm; total organic carbon, <5 ppb) and stored with Chelex (100 sodium form, Sigma-Aldrich) to clean the electrolyte of any metal impurities^74^. H_2_SO_4_ (95–98%, Sigma-Aldrich), H_2_O_2_ (35%, Merck) and KMnO_4_ (99%, Sigma-Aldrich) were used to clean the cells. Argon (5.0 purity, Linde) and CO_2_ (4.5 purity, Linde) were used for purging the electrolytes.

### High-pressure, high-temperature cell set-up

We used an adapted high-pressure, high-temperature cell from Parr Instruments; this cell has been described in detail in a recent paper^51^. The cell was designed to work up to 140 bar and 200 °C. The cell was used in semicontinuous mode, in which CO_2_ was constantly purged through the cell with a flow rate of 150 ml min^−1^. The high flow rate was to ensure proper mass transport while the electrode was not rotated and the electrolyte was not being stirred. The outlet of the cell was coupled to a condenser and a cooler (Cool-Care, Van der Heijden Labortechnik) to keep all volatile products in the liquid phase in the set-up. The outlet gas was analysed by gas chromatography (GC) every 6 min with an Agilent Micro GC with two thermal conductivity detectors. A combination of a MS7A and a CP-PORABOND Q column was used to detect H_2_, CO and CH_4_, and an Al_2_O_3_ column was used to detect the C_2_ and C_3_ hydrocarbons. For the detection of higher hydrocarbons, a Shimadzu 2014 GC with an RTX-1 column and a flame ionization detector was used because this offered better sensitivity. The inlet of the Shimadzu GC was coupled to the outlet of the Micro GC in this configuration. Both GCs have been calibrated with custom calibration gasses from Messer to determine the retention time of the different products and to quantify these. The gas chromatograms of the different columns and GC systems are shown in Supplementary Figs. 4 and 5. The pressure in the cell was maintained by controlling both the inlet and outlet flow with flow controllers (SLA5850, Brooks Instruments). The temperature was controlled with an electric heating mantle. A homemade two-compartment polyether ether ketone cup was used inside the stainless steel chamber and all metal parts from the thermocouple and reference electrode were covered by Teflon tape. Moreover, the lid of the vessel was covered with Viton rubber to prevent any contamination from the stainless steel.

### General electrochemistry

The homemade polyether ether ketone cup was cleaned prior to experiments by storing in permanganate solution overnight (0.5 M H_2_SO_4_, 1 g l^−1^ KMnO_4_). Afterwards, the cell was rinsed, submerged in a diluted mixture of H_2_SO_4_ and H_2_O_2_ to remove any traces of MnO_4_ and MnO_2_, rinsed again and boiled three times with Milli-Q water. A 1-mm-thick copper wire (99.99%, Mateck) was used as the working electrode. Before experiments, the working electrode was electropolished in 85% H_3_PO_4_ (Suprapure, Merck) by applying +3 V versus a graphite counter-electrode for 20 s and subsequently rinsed with Milli-Q water. Due to the large difference in current at the various conditions used, four different sized wires were used to ensure the current stays between 10 mA (for product detection) and 200 mA (for stability of the system) during the experiment. The areas of different working electrodes were normalized using their double-layer capacitance, measured from −0.3 to 0.2 V versus SHE at scan rates from 200 to 1400 mV s^−1^ (ref. ^75^). The areas of the electrodes can be found in Supplementary Table 1.

During experiments, a DSA counter-electrode (Magneto) was separated from the working and reference electrode by a PiperION membrane (Versogen)^76,77^. The experiments were performed with a Gamry Interface 1010B potentiostat, and current interrupt^78^ was used to correct for the ohmic drop. Experiments were performed for 60 or 90 min at constant potential. Gaseous products were determined every 6 min with GC as described above, while liquid products were determined afterwards by sampling the 65-ml catholyte and analysing this using high-performance liquid chromatography (Shimadzu) with an Aminex HPX-87H column (Bio-rad). During experiments the pressure was maintained within an error of 0.1 bar, while temperature was maintained within an error of 2 °C. Before reaching the conditions used for the experiment (which typically takes up to 1 h), the working electrode was always under potential control at a potential of −0.8 V versus Ag/AgCl.

### Reference electrode corrections

In this study, all potentials are reported versus SHE(*T*) — that is, the standard hydrogen electrode at a given temperature *T*. During experiments, an Ag/AgCl electrode (Ultradeg, 0.1 M KCl, Corr Instruments) was used as a reference electrode. However, with changing temperature, one should make several corrections to convert the potential from Ag/AgCl to SHE. The Nernst equation is used to determine the shift of the Ag/AgCl reference electrode against SHE:

1 $${E}_{{\rm{Ag}}/{\rm{AgCl}}}={E}_{{\rm{Ag}}/{\rm{AgCl}}}^{0}(T)-\,\frac{{\rm{ln}}(10)\times {RT}}{{nF}}\times \log ({a}_{{{\rm{Cl}}}^{-}}(T))$$

where *E*^0^ is the standard potential, *R* is the gas constant, *T* is the temperature, *n* is the number of electrons, *F* is Faraday’s constant and ${a}_{{\mathrm{Cl}}^{-}}$ is the activity of the chloride anion. The standard potential of Ag/AgCl versus SHE is a function of temperature and can be calculated using the following formula^79^:

2 $${E}_{{\rm{Ag}}/{\rm{AgCl}}}^{0}(T)=-0.111658+0.011134\times T-0.001757268\times T\times {\rm{ln}}(T)$$

The temperature dependence of the activity of the chloride ions can be determined from a formula from Bogaerts et al.^80^:

3 $${a}_{{\mathrm{Cl}}^{-}}\left(T\right)=\exp \left[\log \left({a}_{{\mathrm{Cl}}^{-}}\left(25\,^\circ {\rm{C}}\right)\right)-\frac{\sqrt{{I}_{{\mathrm{Cl}}^{-}}}}{1+\sqrt{{I}_{{\mathrm{Cl}}^{-}}}}\times \left({A}_{\gamma }^{T}-{A}_{\gamma }^{25\,^\circ {\rm{C}}}\right)\right]$$

where ${a}_{{\mathrm{Cl}}^{-}}$ is the activity of the chloride anion at room temperature^81^, *I*_Cl_ is the ionic strength of the chloride anion and *A*_*γ*_ is the Debye–Huckel parameter^80^.

With these formulas, the shift between an Ag/AgCl reference and SHE could be determined at different temperatures as shown in Supplementary Table 2. Although the SHE potential is defined to be zero at all temperatures, there is a difference in potential between SHE(*T*) and SHE (25 °C)^82^. One could correct for this difference too, but we decide to limit the corrections to the necessary corrections and therefore report on a SHE(*T*) scale. In previous studies we reported on the RHE(*T*) scale^14,31,32,83^, but in this study the potential could not be reported versus RHE because the pH should be known to convert SHE to RHE. However, it is impossible to measure the exact pH of the electrolyte at elevated CO_2_ pressures and temperatures.

### Raman experiments

Raman spectroscopy measurements were carried out to determine the formation of coke during the electrochemical CO_2_RR on copper. The measurements were carried out ex situ after the reaction at both 25 and 125 °C. A confocal spectrometer (Witec Alpha300R) was used with a 457-nm excitation wavelength laser. A 100× times magnification objective was used for spectra collection. The laser power was kept below 2 mW to prevent sample damage. All measurements were performed under ambient conditions at room temperature. Optical images of the electrode were recorded using an optical camera fitted to the Raman microscope set-up.

### Computational methods

All calculations were performed using the spin-polarized DFT approach using the Vienna Ab initio Simulation Package (VASP)^84,85^. The core electrons were treated using the projector-augmented-wave pseudopotential^86^, and the Perdew–Burke–Ernzerhof exchange-correlation functional of the generalized gradient approximation was used to describe electron interactions^87^. For the (100) surface, a 4 × 4 surface unit cell with four layers oriented perpendicular to the flat planes was used, while a 2 × 3 surface unit cell with three layers perpendicular to the terrace planes was used for the (211) surface, keeping the two bottom layers fixed. A vacuum space of 15 Å in the *z* direction was created to prevent interactions between periodic images. The cut-off energy was set to 450 eV, and the convergence criterion for the force on each atom was relaxed to below 0.05 eV Å^−1^. Van der Waals interactions were accounted for using the empirical correction according to Grimme’s scheme^88^. The reciprocal space was sampled with 3 × 3 × 1 *k*-points for the (100) surface and 2 × 3 × 1 *k*-points for the (211) surface. The projected COHP^89–92^ was utilized to investigate the bonding strength of C–O in*COH and *CHO. Transition states were calculated using the climbing image nudged elastic band approach^93^. The effects of the solvent were taken into account using an implicit solvation model^94,95^. A constant-potential method was used to calculate the PCET process^96,97^. Computational details and further explanations can be found in the Supplementary Information.

## Supplementary information

Supplementary Information Supplementary Figs. 1–17, Supplementary References, Supplementary Tables 1–7, and Supplementary Notes 1–6.

Supplementary Data 1 Structures coordinates of DFT calculations

## Data Availability

Electrochemical data can be found in Supplementary Tables 3 and 4. The atomic coordinates of the optimized computational models used in the electronic structure calculations can be found as Supplementary Data. Any additional data can be provided on request from the corresponding author.
