# Supplementary material for: CO2 electroreduction on Cu operates via an alternative chain growth mechanism to form C–C bonds at elevated temperature and pressure
Source: Nat Catal. 2025 Dec 12;8(12):1338–47. doi: 10.1038/s41929-025-01451-1 (PMC12727537; doi:10.1038/s41929-025-01451-1)
Supplement: Supplementary file 1 — Supplementary Figs. 1–17, Supplementary References, Supplementary Tables 1–7, and Supplementary Notes 1–6. [file 41929_2025_1451_MOESM1_ESM.pdf]

nature catalysis

Supplementary information https://doi.org/10.1038/s41929-025-01451-1

CO_2 electroreduction on Cu operates via an alternative chain growth mechanism to form C–C bonds at elevated temperature and pressure

In the format provided by the authors and unedited

Table of contents:

Working and reference electrode 2

Unstable currents at elevated temperatures 2

Switching mechanism at high pressure and high temperature 3

XPS and Raman spectra 8

DFT calculations 10

References 12

Working and reference electrodes

Supplementary Table 1: Geometric electrode area based on double layer capacitance measurements. A, B, C and D are 1 mm Cu wires of different lengths, while E is a reference Cu disk (8mm diameter) with known geometric surface area

Electrode	A	B	C	D	E
C (μF)	252	139	44.3	21.1	27.8
Area (cm^2)	4.556	2.513	0.8010	0.3815	0.5027

Electrode A was used for experiments at 25 °C and 50 °C, and at -1.2 V vs SHE and 125 °C,

Electrode B was used for experiments at -1.3 V vs SHE and 100 to 125 °C

Electrode C was used for experiments at -1.3 V vs SHE and 150 °C

Electrode D was used for experiments at -1.5 V vs SHE above 75 °C

Supplementary Table 2: Shift of the Ag/AgCl reference with 0.1 M KCl at different temperatures, compared to SHE(T), calculated as described in the experimental

T (°C)	25	50	75	100	125	150
$E^{Ag/AgCl}$ (mV vs SHE)	230.3	214.5	195.9	174.6	151.1	125.5

Unstable currents at elevated temperatures

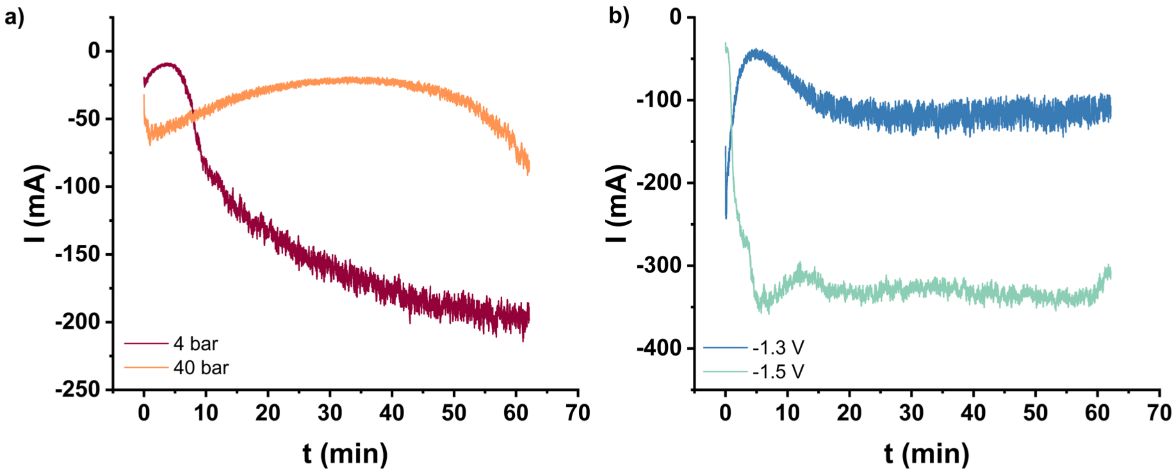

Supplementary Figure 1: Current as function of time for a) 4 and 40 bar at -1.5 V vs SHE at 75 °C and b) 24 bar at 125 °C at -1.3 V and -1.5 vs SHE. This illustrates that at elevated temperatures the current is not stable, especially at low pressures as seen in a). In b) a typical current profile at 125 °C and -1.3 V is observed, where the current first decreases in the first 20 minutes and then increases again after which it stabilizes. It also shows the instability at higher overpotentials, where even the smallest electrode goes to currents above -300 mA, while at the start it was only -30 mA.

Switching mechanism at high pressure and high temperature

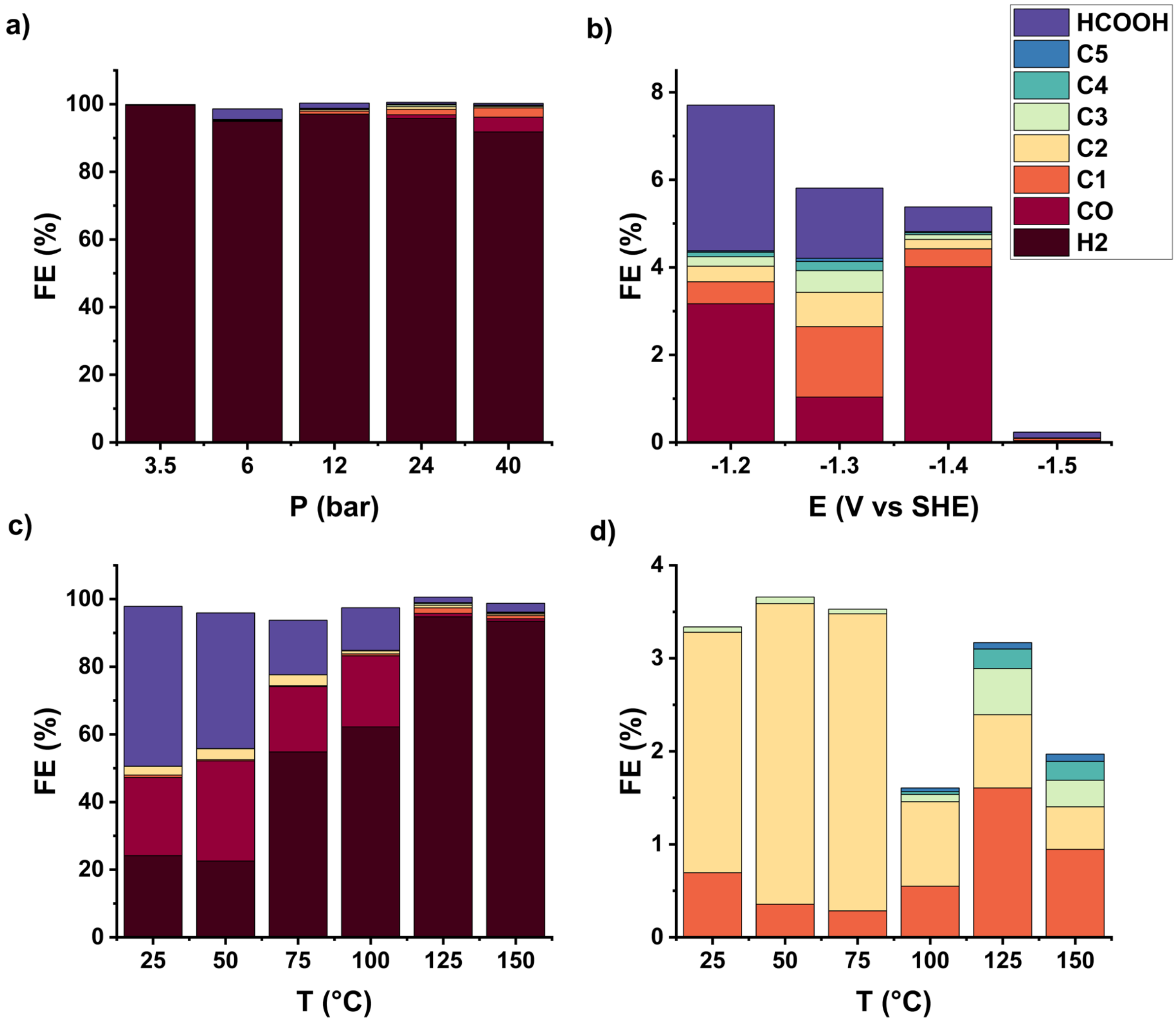

Supplementary Figure 2: Faradaic efficiencies for the CO2RR reduction as function of a) pressure at 125 °C and -1.3 V vs SHE b) potential at 24 bar and 125 °C and c and d) temperature at 24 bar and -1.3 V vs SHE. a) and c) show total FE. b) shows the CO2RR products with the remaining FE being H2 and d) shows only the hydrocarbons.

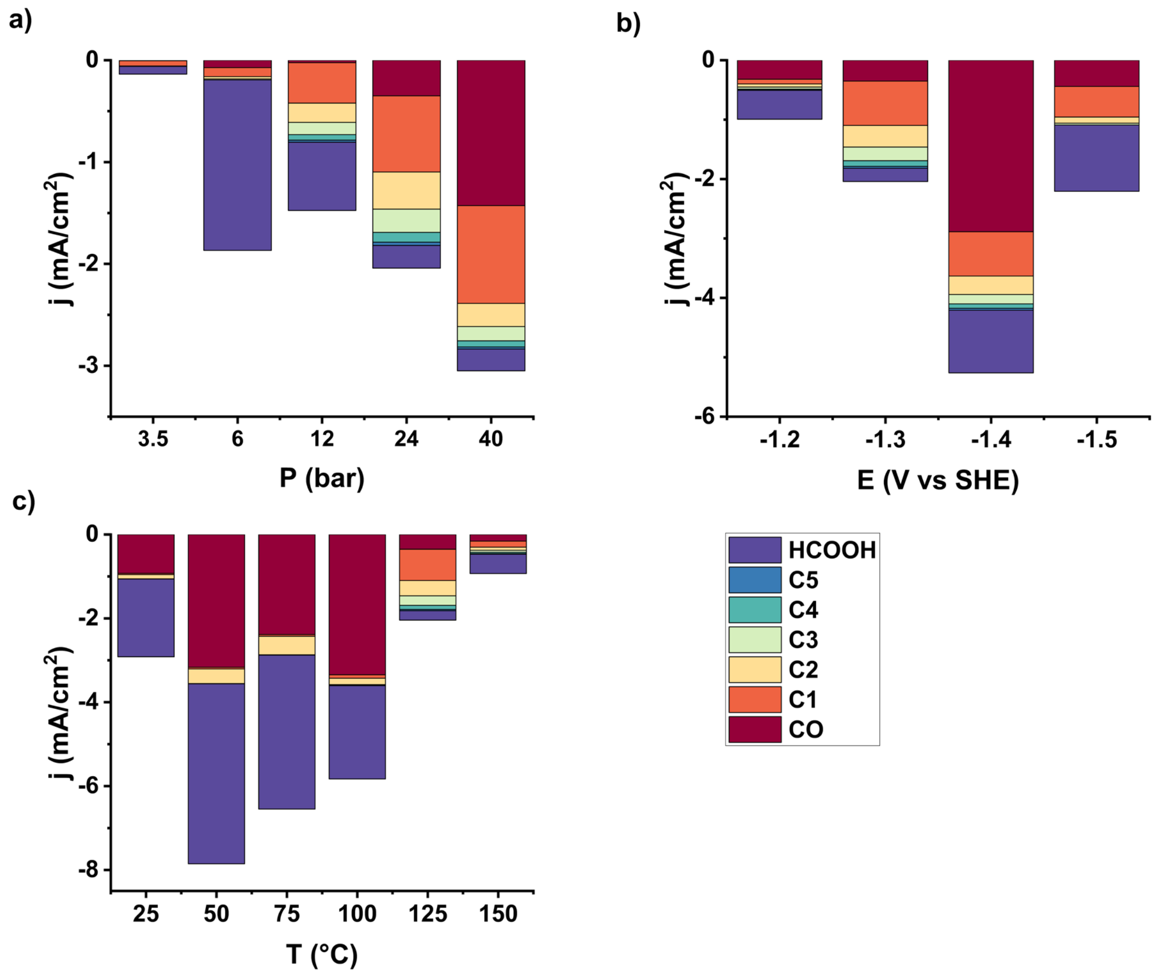

Supplementary Figure 3: Partial current density for the CO2RR products as function of a) pressure at 125 °C and -1.3 V vs SHE b) temperature at 24 bar and -1.3 V vs SHE c) potential at 24 bar and 125 °C.

Supplementary Table 3: Faradaic efficiencies for the CO2RR products on a Cu wire as a function of temperature, pressure and potential. Data is plotted in Figure 1 and S2

T (°C)	P (bar)	E (V vs SHE)	FE (%)									
			H_2	CO	CH_4	C_2H_6	C_2H_4	C_3H_8 C_3H_6	C_4H_{10} C_4H_8	C_5H_{12} C_5H_{10}	HCOOH	CH_3COOH
25	24	-1.3	24.1	23.2	0.7	-	2.6	-	-	-	47.2	2.7
50	24	-1.3	22.5	29.6	0.4	-	3.2	-	-	-	40.1	0.5
75	24	-1.3	54.8	19.3	0.3	0.0	3.2	0.1	0.0	-	16.1	0.4
100	24	-1.3	62.2	21.0	0.5	0.1	0.9	0.1	0.0	0.0	12.6	0.1
125	24	-1.3	95.8	1.1	1.6	0.5	0.3	0.5	0.2	0.1	0.5	0.0
150	24	-1.3	93.5	0.7	1.0	0.1	0.4	0.3	0.2	0.1	2.6	0.7
125	3.5	-1.3	99.4	-	0.1	-	-	-	-	-	0.1	0.1
125	6	-1.3	95.0	0.3	0.2	0.0	0.1	0.0	-	-	3.1	0.1
125	12	-1.3	97.0	0.1	0.9	0.2	0.2	0.3	0.1	0.0	1.5	0.1
125	40	-1.3	91.8	4.4	2.7	0.2	0.2	0.3	0.1	0.0	0.5	0.1
125	24	-1.2	92.0	3.2	0.1	0.2	0.1	0.2	0.1	0.0	3.3	0.1
125	24	-1.4	94.2	3.1	0.1	0.1	-	0.1	0.1	0.0	0.5	-
125	24	-1.5	99.7	0.1	0.0	0.0	-	0.0	-	-	0.1	-

Supplementary Table 4: More detailed Faradaic efficiencies for the C3+ products on a Cu wire as a function of temperature, pressure and potential. The accuracy of some of the data is limited as the peaks in the gas chromatogram for some products are very small. Only to distinguish between different products two decimals are used in this table, but one should be aware of the limitations in accuracy. A "-" means the product is not detected while 0.00 means the product has been detected but the FE is too low to be significant in the table.

T (°C)	P (bar)	E (V vs SHE)	FE (%)								
			Propane	Propene	Iso butane	Butane	Butene	Iso butene	Iso pentane	Pentane	1-Pentene
25	24	-1.3	-	-	-	-	-		-	-	-
50	24	-1.3	-	-	-	-	-		-	-	-
75	24	-1.3	-	0.05	0.00	0.00	0.01		-	-	-
100	24	-1.3	-	0.08	0.00	0.01	0.02	0.00	0.01	0.01	0.02
125	24	-1.3	0.05	0.45	0.03	0.07	0.10	0.01	0.01	0.02	0.04
150	24	-1.3	0.03	0.26	0.04	0.07	0.09	0.00	0.01	0.02	0.04
125	3.5	-1.3	-	-	-	-	-		-	-	-
125	6	-1.3	-	0.02	-	-	-		-	-	-
125	12	-1.3	0.03	0.24	0.02	0.04	0.05	0.00	0.01	0.01	0.02
125	40	-1.3	0.04	0.25	0.02	0.04	0.06	0.00	0.01	0.01	0.02
125	24	-1.2	0.02	0.19	0.02	0.04	0.04	0.00	0.01	0.01	0.02
125	24	-1.4	0.00	0.10	0.01	0.02	0.03	0.00	0.00	0.01	0.01
125	24	-1.5	-	0.00	-	-	-		-	-	-

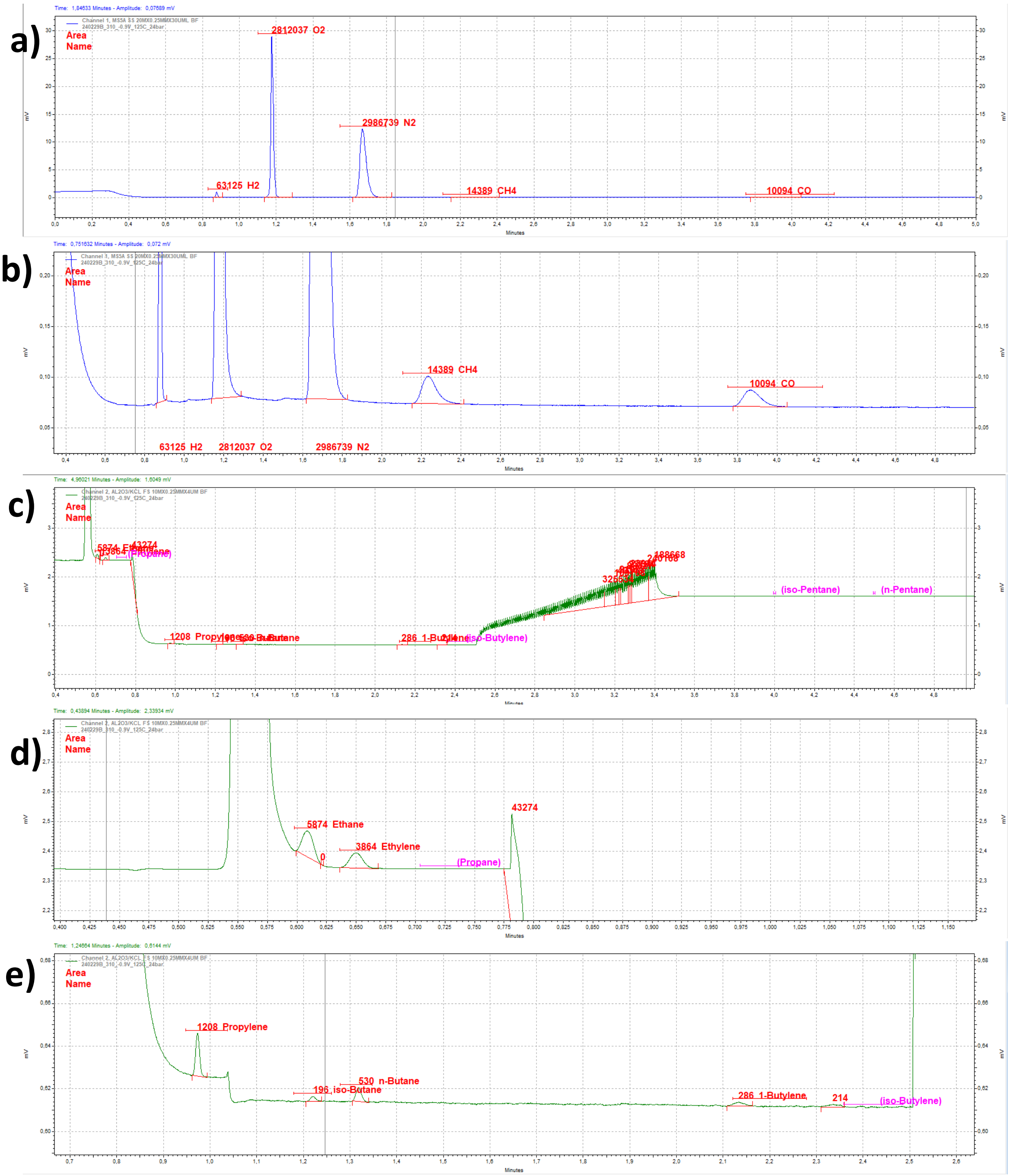

Supplementary Figure 4: Examples of gas chromatograms. a) and b) show the chromatogram for the CP-PORABOND Q column in a Micro-GC from Agilent to detect H2, CO and CH4, where b) is a zoom-in from a). c) d) and e) show the chromatogram for the Al2O3 column in a Micro-GC from Agilent to detect C2 and C3 hydrocarbons, where d) and e) are zoom-ins from c).

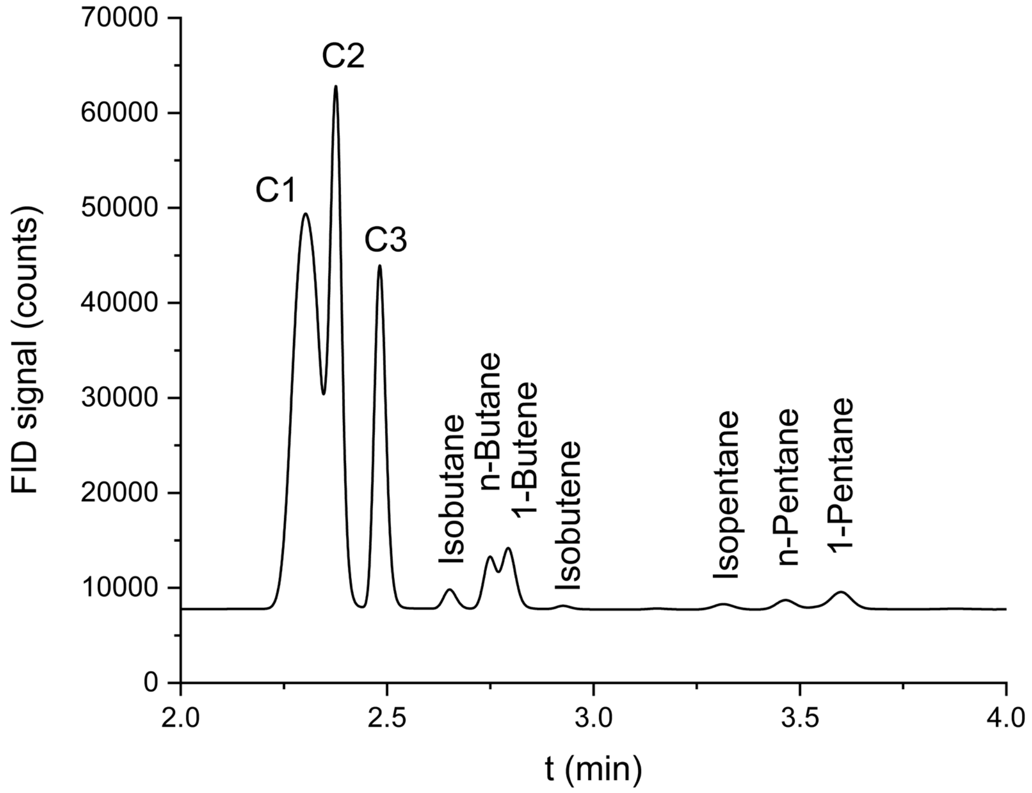

Supplementary Figure 5: Examples of a gas chromatogram showing the chromatogram for the RTX-1 column in a Shimadzu 2014 GC to detect C4 and C5 hydrocarbons. The Shimadzu 2014 GC was used to detect the C4 and C5 hydrocarbons and optimized for this as this was more sensitive than the Micro-GC from Agilent, in which we were only able to detect up to C4 as can be seen in figure e). Because the Shimadzu column was optimized to observe the C5 hydrocarbons, the peaks for C1-C3 hydrocarbons get convoluted, therefore, these were determined with the Micro-GC in Supplementary Figure 4d and 4e.

Supplementary Table 5: Chain growth probability as function of temperature, pressure and potential, as obtained by fitting the product distribution with the Anderson-Schulz-Flory distribution

Temperature (°C)	Pressure (bar)	Potential (V vs SHE)	Chain growth probability
75	24	-1.3	0.22 ±0.02
100	24	-1.3	0.30 ±0.02
125	24	-1.3	0.33 ±0.01
150	24	-1.3	0.41 ±0.01
125	12	-1.3	0.35 ±0.01
125	40	-1.3	0.30 ±0.01
125	24	-1.2	0.41 ±0.01
125	24	-1.4	0.34 ±0.01
125	24	-1.5	0.31 ±0.01

XPS and Raman spectra

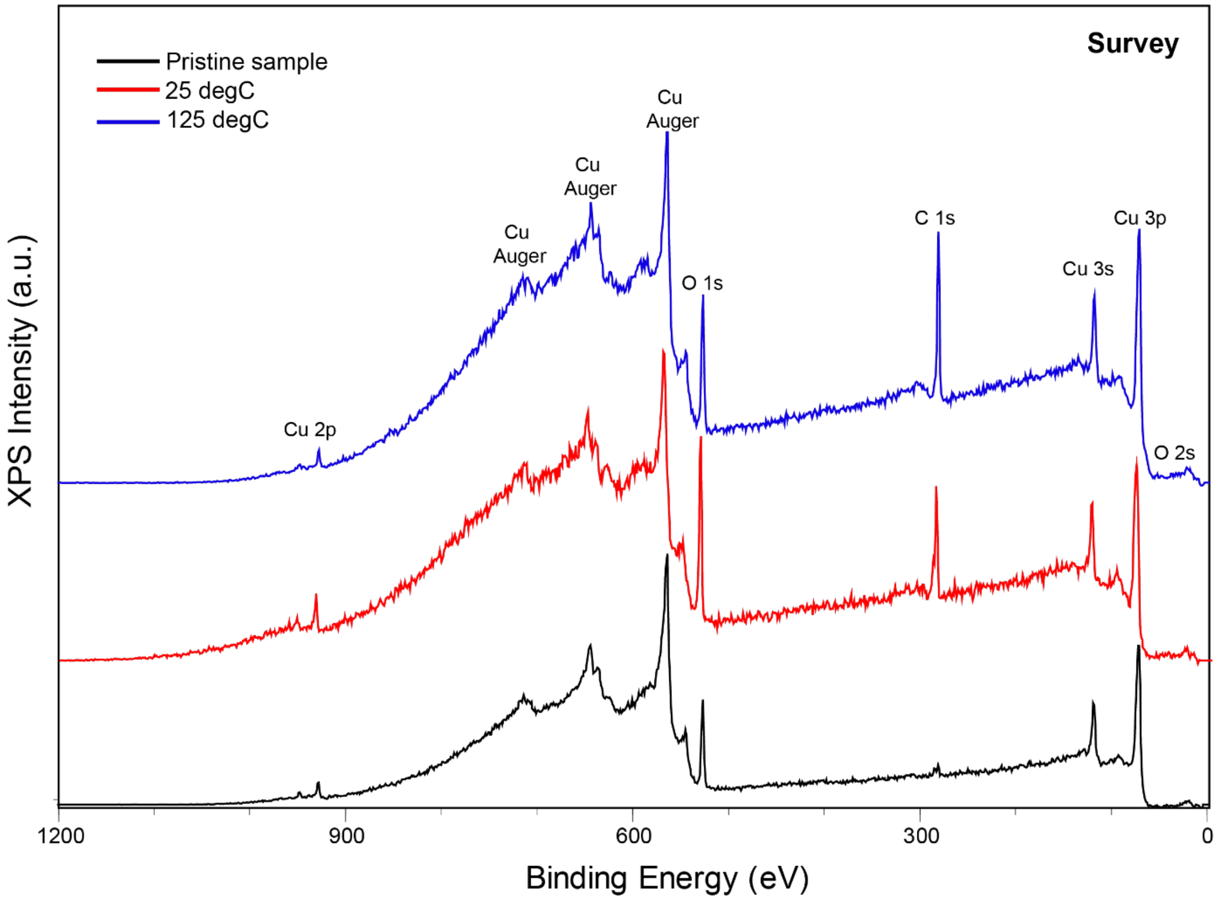

Supplementary Figure 6: XPS spectra of electropolished Cu and Cu after CO2RR in 0.2 M $KHCO_3$ at 25 and 125 °C. The XPS spectra were acquired using a lab source NAPXPS setup[1], equipped with SPECS PHOIBOS 150 analyzer and XR-MF X-ray source (Al K-alpha) with a 0.3mm x-ray spot. The spectra were acquired with a pass energy of 20 eV using the instrument software (SPECS Lab Prodigy). Data analysis and background correction was performed using CASA XPS software. A Shirley background correction was performed on the Cu 2p spectra while a linear background was subtracted from the survey spectra. The Cu 2p spectra are normalized to the Cu 2p 3/2 peak.

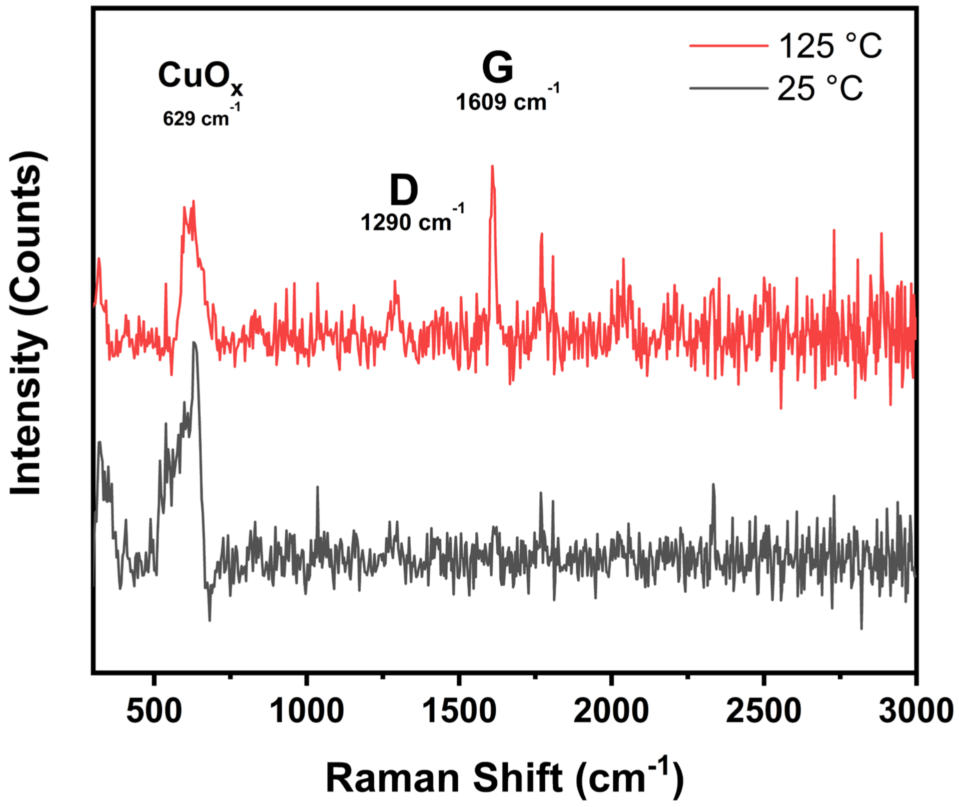

Supplementary Figure 7: Extended Raman spectrum after CO2RR on Cu in 0.2 M $KHCO_3$ at 25 and 125 °C showing the formation of coke on the electrode at elevated temperatures and showing the Cu oxide signal for both samples.

Supplementary notes

Density Functional Theory calculations

Note 1: The adsorption energies (E_{ads}) were calculated as the energy difference between the optimized surface with the adsorbed species (E_{tot}) and the sum of the energies of the optimized clean surface and the isolated molecules (E_{mol} + E_{slab}), which is defined by

$$E_{ads} = E_{tot} - E_{slab} - E_{mol} \qquad (1)$$

The energy of isolated COH and CHO molecules was referenced to the energy of CO and 1/2 H_2.

Note 2: An implicit electrolyte model implemented in VASPsol code [2,3] was used, featuring a Debye length of 3.0 Å (corresponding to a 1 M ionic strength) and a dielectric constant of 80 for the aqueous solution.

Note 3: For the calculation of the reaction rate, the Arrhenius equation was used with a pre-exponential factor of k_BT/h as a simplification:

$$k = \frac{k_B T}{h} \exp\left(-\frac{\Delta G_a}{k_B T}\right) \qquad (2)$$

where k_B is the Boltzmann constant, T is the temperature, and h is Planck's constant, and ΔG_a is the free energy barrier, for which we use the calculated values. Even if this pre-exponential factor is (far) off the real value for a surface reaction, it is important that the values are the same for reactions of the same type.

Note 4: Constant-potential calculations were carried out by simultaneously optimizing the atomic coordinates and the system's electron number to match the desired applied potential on the standard hydrogen electrode (SHE) scale. This approach ensures consistency with the electrochemical conditions being studied[4-6]. The implicit solvent model, based on the linear polarizable continuum model (PCM), was used for electrolyte simulations and implemented through VASPsol [2,3]. To accurately represent the aqueous environment, the solvent's relative permittivity was set to 80 and a Debye length of 3 Å was used. Additionally, the surface tension parameter was set to zero to exclude cavitation energy contributions. For charged systems, a counter-ion charge distribution, modeled using the generalized Poisson-Boltzmann method, was incorporated into the implicit solvent region.

The applied potential on the SHE scale was determined using

$$U_{SHE} = (-\Phi_{SHE} - \Phi)/e = (-4.6\ \text{eV} - \Phi)/e \qquad (3)$$

where Φ_{SHE} and Φ are the absolute potentials for SHE and the electrode/electrolyte interface, respectively. The electrochemical nudged elastic band (eNEB) method[6] was utilized to identify transition states under constant-potential conditions.

Note 5: The proton-coupled electron transfer (PCET) process was modeled using two types of proton donors: an H_8O_4 cluster to represent water as the proton source, and an $H_9O_4^+$ hydronium–water complex (formed by a solvated H^+ with three explicit water molecules) to represent H^+ as the proton source. This setup effectively captures the localized bonding interactions between the adsorbate, surface, and solvent molecules. To account for long-range electrostatic effects, we utilized the implicit solvation model implemented in VASPsol [2,3]. This hybrid approach balances computational efficiency with reliability, providing a robust framework for studying the PCET mechanism [8].

Note 6: The free energies shown in the Surface Pourbaix diagrams (G_{SP}) were computed based on the method described by Supplementary Ref. 9-11:

$$G_{SP} = G_{bare} + mG_{H_2O} - G_{tot} - (2m - n)(1/2G_{H_2} - U_{SHE} - 2.303k_BT*pH) \qquad (4)$$

where G_{bare} is the total free energy of a bare surface, G_{H_2O} is the free energy of H_2O, G_{tot} is the total free energy of the surface with adsorbate, G_{H_2} is the total energy of H_2. m and n represent the numbers of oxygen and

hydrogen atoms adsorbed on the surface, respectively. k_B is the Boltzmann constant, and T is the temperature.

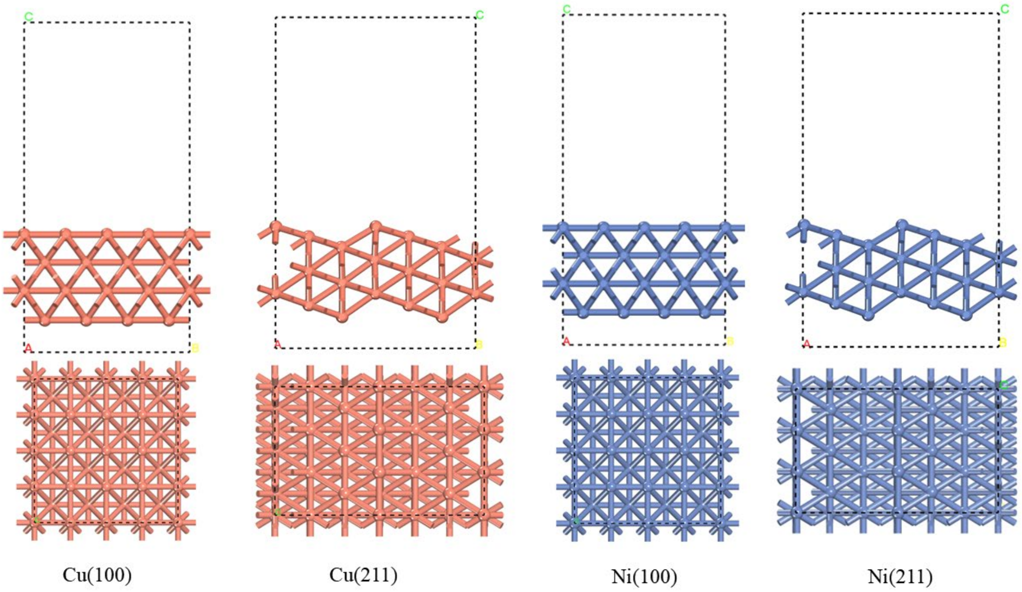

Supplementary Figure 8: (100) and (211) facets of the Cu and Ni from side and top perspectives.

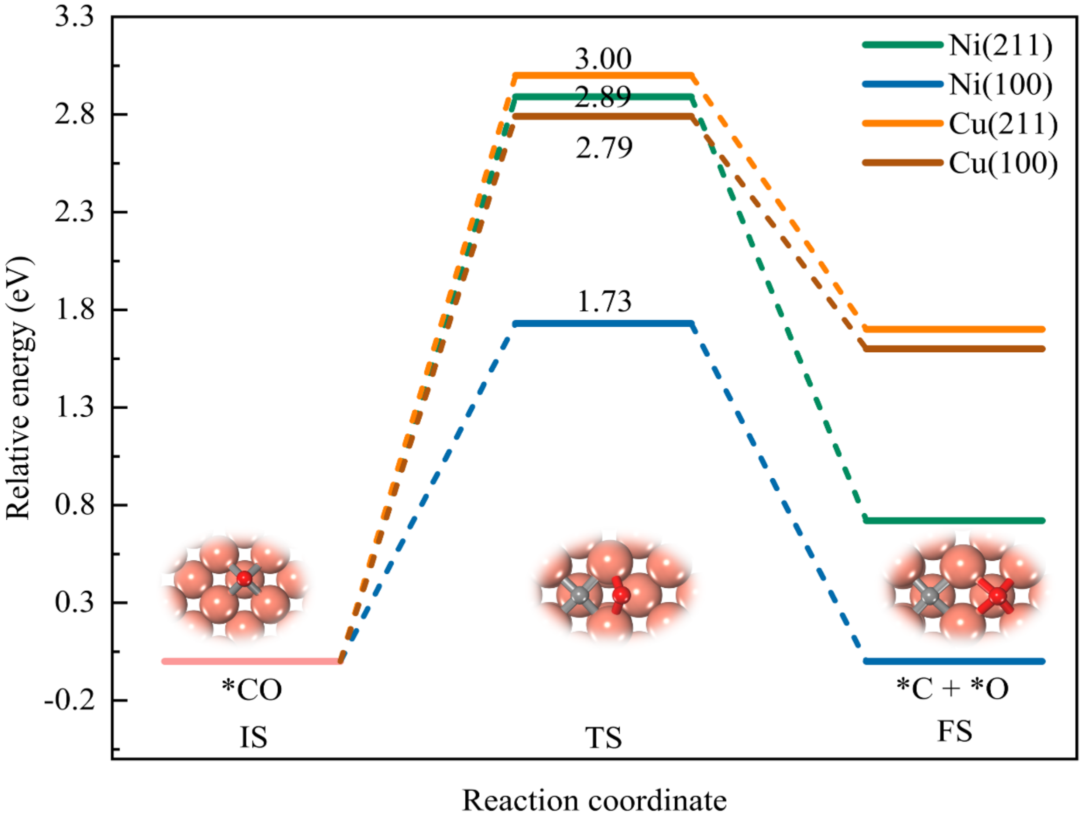

Supplementary Figure 9: Energy profile for initial states, transition states (TSs) and final states of *CO dissociation on Cu (100), Cu (211), and Ni (100), Ni (211) surfaces. Representative structures of the initial state, transition state, and final state of *CO dissociation on the Cu (100) surface are also shown.

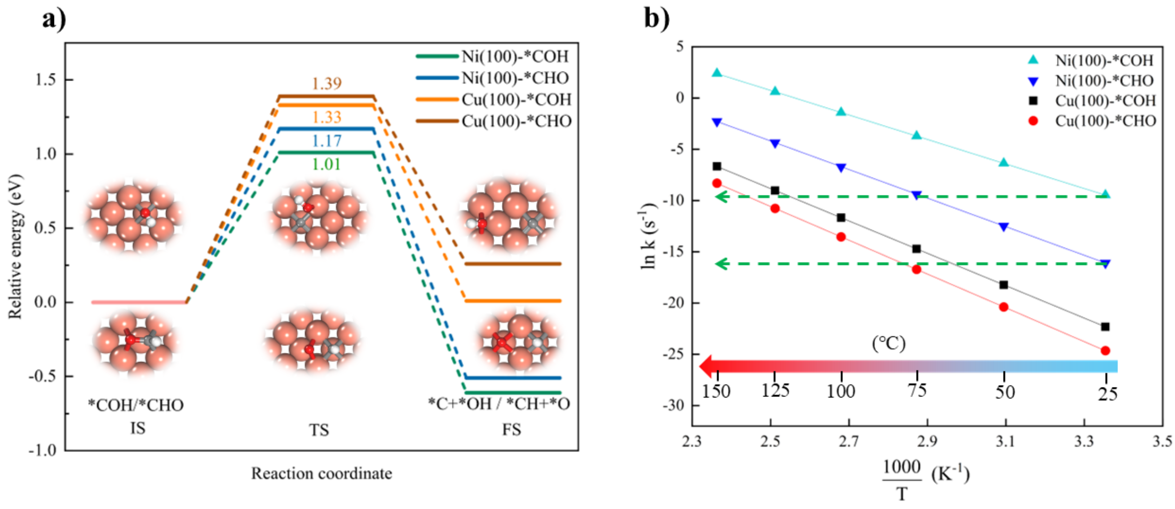

Supplementary Figure 10: *COH/*CHO dissociation on Cu (100), and Ni (100) surface a) Energy profiles for initial states, transition states (TSs) and final states of *COH/*CHO dissociation on Cu (100), and Ni (100) surface. Representative structures of the initial states, transition states and final states of *COH/*CHO dissociation on the Cu (100) surface are also shown; **b)** The dissociation rate constant (k) of *COH/*CHO as a function of temperature on Cu (100), and Ni (100) surface. The green line represents the decomposition rate of *COH/*CHO on the Ni surface at 25 ^{0}C.

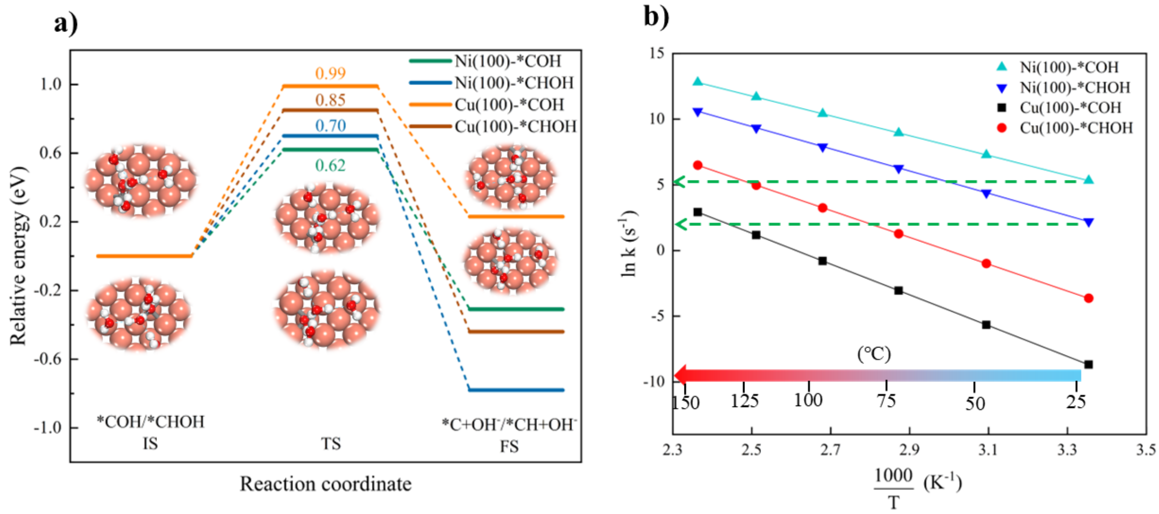

Supplementary Figure 11: *COH/*CHOH proton-coupled electron transfer process on Cu (100), and Ni (100) surface a) Profiles for initial states, transition states (TSs) and final states of *COH/*CHOH proton-coupled electron transfer process on Cu (100), and Ni (100) surface at U = −1.3 V versus SHE. Representative structures of initial, transition and final states of *COH/*CHOH proton-coupled electron transfer process on the Cu (100) surface are also shown; b) The variation of proton-coupled electron transfer process rate constants (k) of *COH/*CHOH with temperature on Cu (100) and Ni (100) surfaces. The green line represents the proton-coupled electron transfer process rates of *COH/*CHOH on the Ni surface at 25 °C

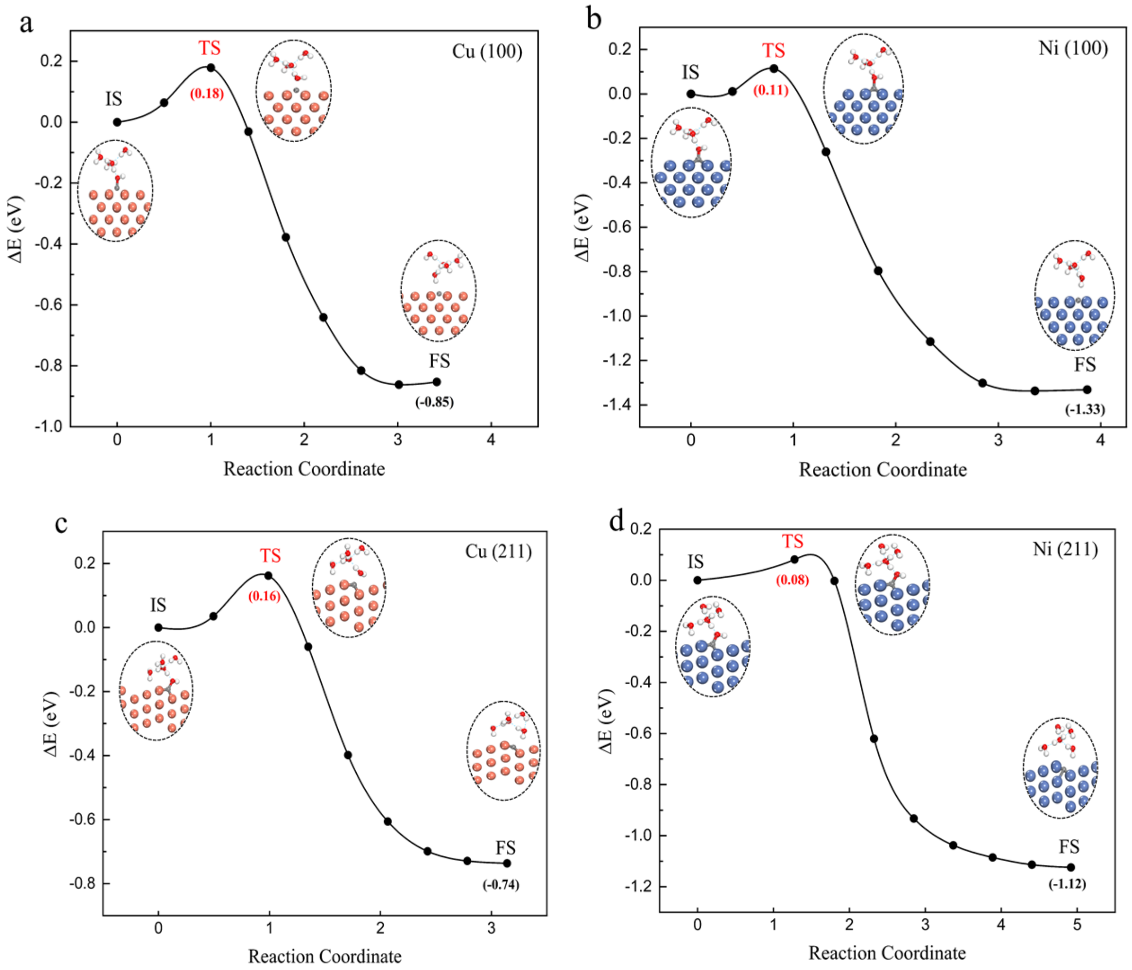

Supplementary Figure 12: Calculated energy profiles using the constant potential method for the conversion from *COH to *C during the PCET process over (a) Cu (100), (b) Ni (100), (c) Cu (211), and (d) Ni (211). 7 images were used for the transition state calculations. IS denotes the initial state, TS represents the transition state, and FS indicates the final state.

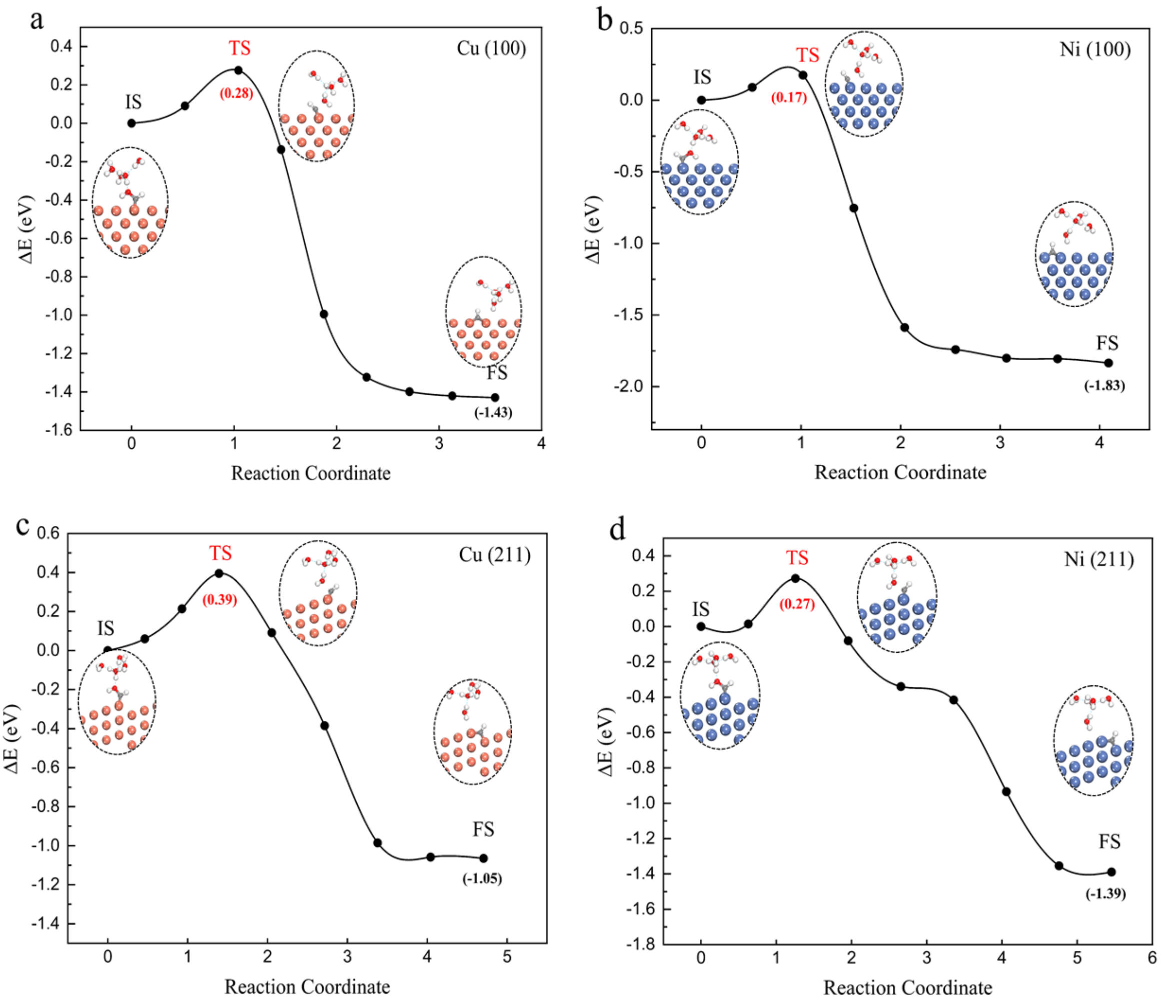

Supplementary Figure 13: Calculated energy profiles using the constant potential method for the conversion from *CHOH to *CH during the PCET process over (a) Cu (100), (b) Ni (100), (c) Cu (211), and (d) Ni (211). 7 images were used for the transition state calculations. The IS denotes the initial state, TS represents the transition state, and FS indicates the final state.

Supplementary Table 6 Comparison of reaction barriers (Ea) and reaction energy changes (ΔE) for CHx species formation on clean Cu and partially oxidized Cu surfaces (1O denotes the addition of one O atom to the clean Cu surface to simulate oxidation).

Elementary Step	Cu (100)		1O-Cu (100)		Cu (211)		1O-Cu (211)	
	Ea(eV)	ΔE(eV)	Ea(eV)	ΔE(eV)	Ea(eV)	ΔE(eV)	Ea(eV)	ΔE(eV)
*CO→*C+*O	2.79	1.60	3.40	2.12	3.00	1.83	3.86	1.69
*COH→*C+*OH	1.33	0.01	1.49	0.12	0.99	-0.11	1.22	0.29
*CHO→*CH+*O	1.39	0.26	1.93	0.80	1.48	0.45	2.39	0.61
*COH+H_2O→ *C+H_2O+OH^-	0.99	0.23	1.14	0.34	1.01	0.38	1.04	0.41
*CHOH+H_2O→ *CH+H_2O+OH^-	0.85	-0.44	1.29	-0.19	1.28	-0.06	1.50	0.12
*COH+H^+→*C+H_2O	0.18	-0.85	0.29	-0.72	0.16	-0.74	0.20	-0.69
*CHOH+H^+→*CH+H_2O	0.28	-1.43	0.39	-1.23	0.39	-1.05	0.51	-0.89

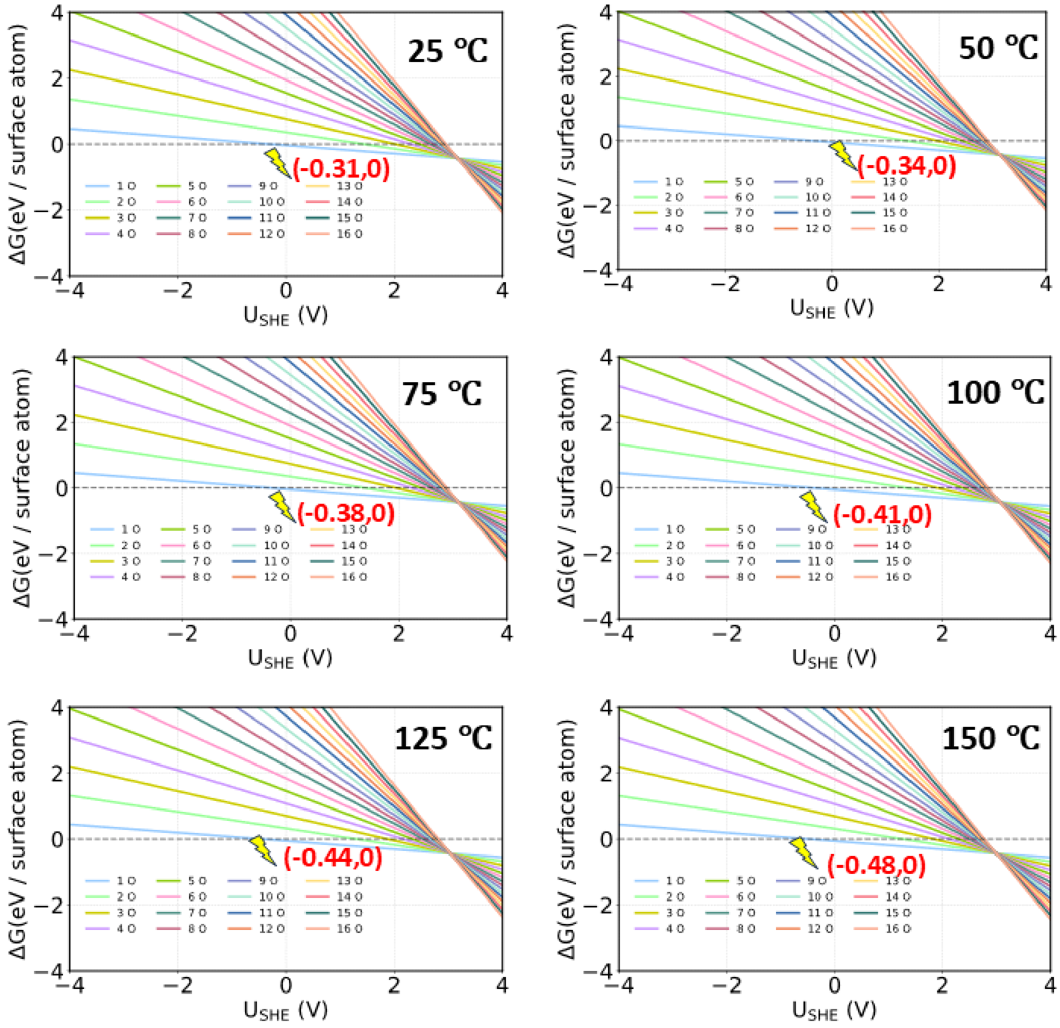

Supplementary Figure 14: Surface Pourbaix diagram for Cu (100). The x-axis in parentheses indicates the initial potential at which the surface is occupied by the first oxygen atom.

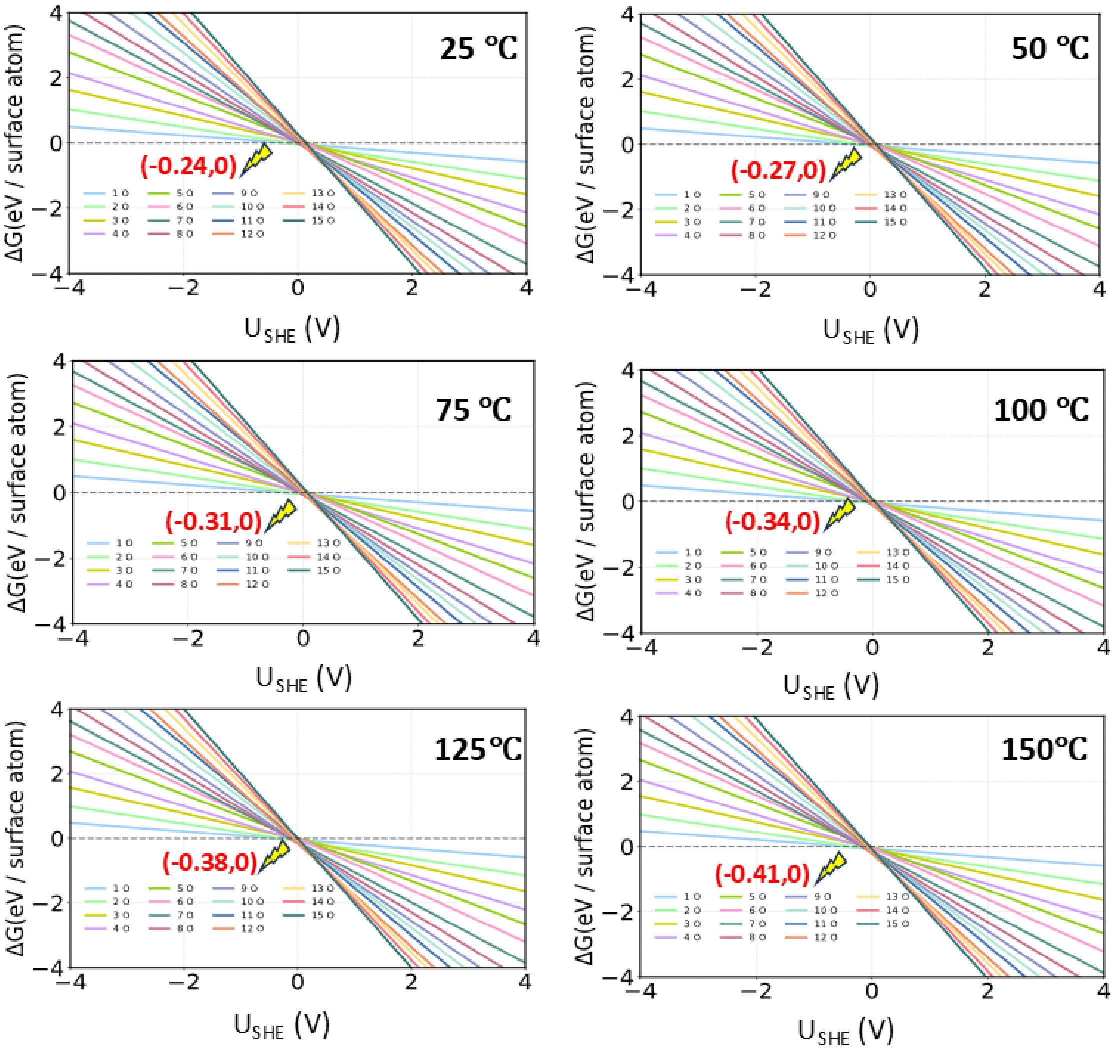

Supplementary Figure 15: Surface Pourbaix diagram for Cu (211). The x-axis in parentheses indicates the initial potential at which the surface is occupied by the first oxygen atom.

Supplementary Table 7 Energy barriers (Ea) and reaction energy changes (ΔE) for $*CH_2$ species coupling with possible C1 species on Cu (100) and Cu (211) surfaces.

Elementary Step	Cu (100)		Cu (211)	
	Ea(eV)	ΔE(eV)	Ea(eV)	ΔE(eV)
$*CH_2+*CO \rightarrow *CH_2CO$	0.72	-0.44	0.59	-0.43
$*CH_2+*C \rightarrow *CH_2C$	0.55	-1.56	0.85	-1.77
$*CH_2+*CH \rightarrow *CH_2CH$	0.41	-1.20	0.24	-1.39
$*CH_2+*CH_2 \rightarrow *CH_2CH_2$	0.05	-1.86	0.03	-1.54
$*CH_2+*CH_3 \rightarrow *CH_2CH_3$	1.25	-0.96	1.47	-0.61

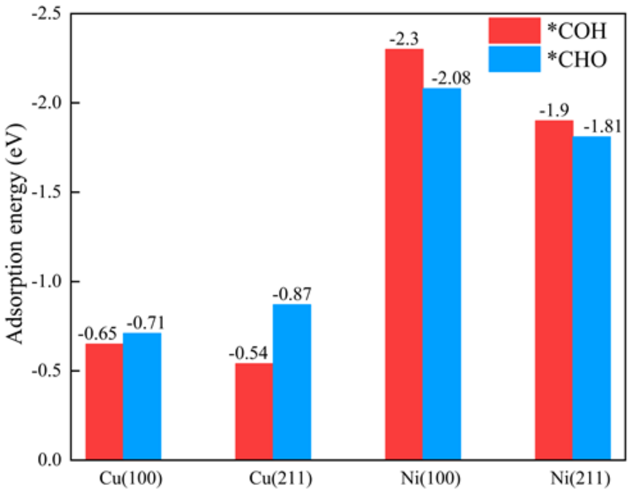

Supplementary Figure 16: Adsorption energies of *COH/*CHO on Cu (100), Cu (211), and Ni (100), Ni (211) surfaces. The energy of isolated COH and CHO molecules was referenced to the energy of CO and 1/2 H_2.

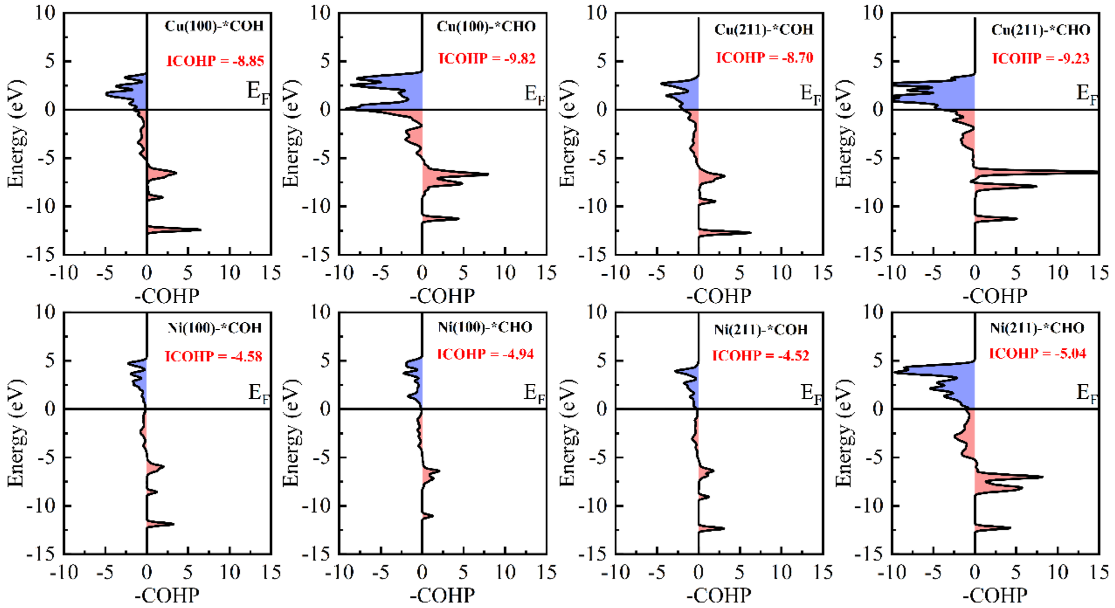

Supplementary Figure 17: Projected crystal orbital Hamilton populations (pCOHP)[12-14] between the C atom and the O atom in *COH/*CHO. More negative ICOHP indicates stronger C-O bond strength. It shows that the C-O bond in *COH is more easy to activate than in *CHO, and that Ni is more suited for activating the C-O than Cu.

Supplementary References

1. Javed, H. et al. A laboratory-based electrochemical NAP-XPS system for operando electrocatalysis studies. *Vacuum* **231**, 113755 (2025).

2. Mathew, K., Sundararaman, R., Letchworth-Weaver, K., Arias, T. A. & Hennig, R. G. Implicit solvation model for density-functional study of nanocrystal surfaces and reaction pathways. *J Chem Phys* **140**, 084106 (2014).

3. Mathew, K., Kolluru, V. S., Mula, S., Steinmann, S. N. & Hennig, R. G. Implicit self-consistent electrolyte model in plane-wave density-functional theory. *J Chem Phys* **151**, 234101 (2019).

4. Osella, S. & Goddard Iii, W.A. CO(2) Reduction to Methane and Ethylene on a Single-Atom Catalyst: A Grand Canonical Quantum Mechanics Study. *J Am Chem Soc* **145**, 21319-21329 (2023).

5. Sundararaman, R., Goddard, W.A., 3rd & Arias, T.A. Grand canonical electronic density-functional theory: Algorithms and applications to electrochemistry. *J Chem Phys* **146**, 114104 (2017)

6. Abidi, N., Bonduelle-Skrzypczak, A. & Steinmann, S.N. Revisiting the Active Sites at the MoS(2)/H(2)O Interface via Grand-Canonical DFT: The Role of Water Dissociation. *ACS Appl Mater Interfaces* **12**, 31401-31410 (2020).

7. Duan, Z. & Xiao, P. Simulation of potential-dependent activation energies in electrocatalysis: Mechanism of O–O bond formation on RuO2. *The Journal of Physical Chemistry C* **125**, 15243–15250 (2021).

8. Re Fiorentin, M. *et al.* Silver Electrodes Are Highly Selective for CO in CO2 Electroreduction due to Interplay between Voltage Dependent Kinetics and Thermodynamics. *J Phys Chem Lett* **15**, 11538–11545 (2024).

9. Hansen, H. A. *et al.* Surface Pourbaix Diagrams and Oxygen Reduction Activity of Pt, Ag and Ni (111) Surfaces Studied by DFT. *Phys Chem Chem Phys* **10**, 3722-3730 (2008).

10. Vinogradova, O. *et al.* Quantifying Confidence in DFT-predicted Surface Pourbaix Diagrams of Transition-metal Electrode–electrolyte Interfaces. *Langmuir* **34**, 12259–12269 (2018).

11. Valdés, Á. *et al.* Oxidation and Photo-oxidation of Water on TiO_2 Surface. *J Phys Chem C* **112**, 9872–9879 (2008).

12. Dronskowski, R. & Blöchl, P. E. Crystal orbital Hamilton populations (COHP): energy-resolved visualization of chemical bonding in solids based on density-functional calculations. *J Phys Chem* **97**, 8617–8624 (1993).

13. Deringer, V. L., Tchougréeff, A. L. & Dronskowski, R. Crystal Orbital Hamilton Population (COHP) Analysis As Projected from Plane-Wave Basis Sets. *J Phys Chem A* **115**, 5461–5466 (2011).

14. Maintz, S., Deringer, V. L., Tchougréeff, A. L. & Dronskowski, R. LOBSTER: A tool to extract chemical bonding from plane-wave based DFT. *J Comput Chem* **37**, 1030–1035 (2016).
